# TMBcat: A multi-endpoint *p*-value criterion on different discrepancy metrics for superiorly inferring tumor mutation burden thresholds

**DOI:** 10.3389/fimmu.2022.995180

**Published:** 2022-09-16

**Authors:** Yixuan Wang, Xin Lai, Jiayin Wang, Ying Xu, Xuanping Zhang, Xiaoyan Zhu, Yuqian Liu, Yang Shao, Li Zhang, Wenfeng Fang

**Affiliations:** ^1^ School of Computer Science and Technology, Xi’an Jiaotong University, Xi’an, China; ^2^ School of Management, Hefei University of Technology, Hefei, China; ^3^ The Ministry of Education Key Laboratory of Process Optimization and Intelligent Decision-Making, Hefei University of Technology, Hefei, China; ^4^ Medical Department, Nanjing Geneseeq Technology Inc., Nanjing, China; ^5^ School of Public Health, Nanjing Medical University, Nanjing, China; ^6^ State Key Laboratory of Oncology in South China, Collaborative Innovation Center for Cancer Medicine, Sun Yat-sen University Cancer Center, Guangzhou, China

**Keywords:** immunotherapy, tumor mutation burden, categorization thresholds, joint efficacy, minimal p-value, between-group difference

## Abstract

Tumor mutation burden (TMB) is a widely recognized stratification biomarker for predicting the efficacy of immunotherapy; however, the number and universal definition of the categorizing thresholds remain debatable due to the multifaceted nature of efficacy and the imprecision of TMB measurements. We proposed a minimal joint *p*-value criterion from the perspective of differentiating the comprehensive therapeutic advantages, termed TMBcat, optimized TMB categorization across distinct cancer cohorts and surpassed known benchmarks. The statistical framework applies to multidimensional endpoints and is fault-tolerant to TMB measurement errors. To explore the association between TMB and various immunotherapy outcomes, we performed a retrospective analysis on 78 patients with non-small cell lung cancer and 64 patients with nasopharyngeal carcinomas who underwent anti-PD-(L)1 therapy. The stratification results of TMBcat confirmed that the relationship between TMB and immunotherapy is non-linear, i.e., treatment gains do not inherently increase with higher TMB, and the pattern varies across carcinomas. Thus, multiple TMB classification thresholds could distinguish patient prognosis flexibly. These findings were further validated in an assembled cohort of 943 patients obtained from 11 published studies. In conclusion, our work presents a general criterion and an accessible software package; together, they enable optimal TMB subgrouping. Our study has the potential to yield innovative insights into therapeutic selection and treatment strategies for patients.

## 1 Introduction

Immune checkpoint inhibitors (ICI) revolutionized cancer therapy (1–4). Research findings demonstrate that tumor mutation burden (TMB) as a stratification biomarker in immuno-oncology helps predict patient prognosis (5, 6). TMB is the number of somatic mutations per megabase (mut/Mb, mainly single-nucleotide variants and short indels). These mutations result in the capacity to generate surface neoantigens that activate T lymphocytes (7), boosting tumor immunogenicity (8, 9). Positive associations between elevated TMB levels and benign ICI prognosis have occurred (10–12). The NCCN guidelines and the FDA prioritized TMB as the recommended test for patients receiving immunotherapy (13, 14).

For clinical decision-making, physicians tend to categorize TMB as a baseline to separate patients into distinct risk groups with varying therapeutic benefits (15). However, due to controversial clinical results, standardized TMB thresholds and the proper number of patient subgroups have not been definitively established. Specifically, i) the available quantile-based benchmarks (e.g., median, quartiles) fail to reflect the underlying biology of TMB and accurately locate the thresholds (16). For example, certain investigations showed that quantile-based TMB cutoffs could not clearly distinguish responders and their prospective clinical benefits (17–19). ii) The typical clinical endpoints for immuno-oncology involve objective tumor response rate (ORR) and time-to-event (TTE), with the TMB biomarker linked to both (20). Inconsistent TMB thresholds arise when statistical studies on the same cohort of patients use different endpoints, leaving clinicians uncertain (21). Instead of basing a general TMB threshold on a single endpoint that discloses only partial therapeutic benefits, a thorough assessment of the disease’s multifaceted efficacy is needed (22, 23).

**Figure 1:**
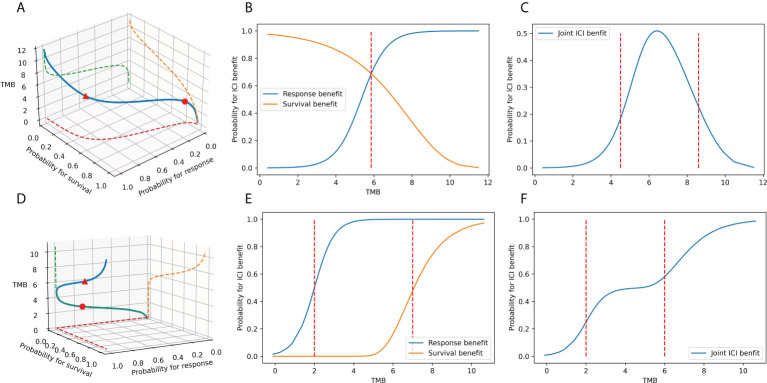
The association between TMB marker and ICI benefits. **(A–C)** When the TMB effects on the response endpoint and survival endpoint have different magnitudes: the association between TMB and ICI clinical benefits in space, the association between TMB and tumor response, the survival benefit in plane, and the association between TMB and joint benefit in the plane. **(D–F)** When the TMB effects on the response endpoint and survival endpoint point in different directions: the association between TMB and ICI clinical benefits in space, the association between TMB and tumor response, the survival benefit in the plane, the association between TMB and joint benefit in the plane.

Furthermore, iii) the effects of different endpoints may vary in magnitude or orientation (24). Such contradiction suggests that the connection between TMB and ICI advantages may not be uniformly distributed and may differ across carcinomas. As shown in **Figures 1B, E**, the associations between TMB and unidimensional outcomes have only one inflection point. When the intensities or directions of the impact of TMB on the distinct endpoints disagree, multiple TMB thresholds permit significantly diverse clinical performances in patient subgroups, either from the three-dimensional space (**Figures 1A, D**) or a joint perspective (**Figures 1C, F**). Clinicians are uncertain about the optimal number of risk groups to stratify patients. Simultaneously, several unobserved common features lead to a natural correlation between tumor response and event time, and the strength of this association varies among regimens and cancer types (25–27). Consequently, the favorable joint probabilities cannot be derived by simply multiplying the probabilities of individual endpoints, which is also a challenge in TMB categorization. Finally, iv) the imprecise nature of TMB markers is another cause of threshold disputes (16). Due to technical restrictions, the variant calling tools will never be perfectly accurate, regardless of the various TMB calculation methodologies (28, 29). TMB is inevitably subject to measurement error. In statistical models that support clinical decision-making, we must account for lessening the instability and bias arising from TMB errors in patient categorization (30).

Therefore, we present TMBcat, a generalized framework based on the minimal joint *p*-value criterion, which can optimize identifying the number of patient subgroups and the corresponding TMB thresholds across all cancers. The framework jointly models multidimensional endpoints while accounting for TMB measurement inaccuracies, yielding the most statistically significant TMB classification based on the minimal *p*-value. The optimized TMB categorization stratifies the patient population significantly and maximizes the discrepancy in clinical performance between subgroups (31). To verify the viability of TMBcat, we collected a cohort of 78 patients with non-small cell lung cancer (NSCLC) and 64 patients with nasopharyngeal carcinoma (NPC) who received ICI treatment. We applied the proposed framework to identify TMB thresholds and revealed novel correlation patterns regarding TMB metrics and immunotherapy efficacy. In some cases, the association between TMB and improved outcomes was non-linear, i.e., the positive correlation was not perfectly straight-line but followed a curved upward pattern varying across regimens or carcinomas, making it more informative to assign patients to multiple categories. Furthermore, we validated these findings in an assembled cohort of 943 patients. The results show that the proposed framework can provide innovative insights into therapeutic refinement for patients. The source code to reproduce the results can be downloaded from https://github.com/YixuanWang1120/JM_TMBcat.

## 2 Materials and methods

### 2.1 A general statistical criterion for TMB categorization

The categorization of TMB indicators facilitates the use of information regarding the relationship between ICI benefits and predictive TMB characteristics in making treatment decisions for clinicians. Therefore, TMB thresholds should distinguish patients with distinct risks. It is, therefore, necessary to establish a general statistical criterion to determine the optimal TMB thresholds and the number of patient subgroups. Our optimization objective is to achieve categorization with the minimum *p*-value, which maximizes the difference in the probabilities of joint ORR&TTE benefit between subgroups. By integrating multidimensional endpoints to model the joint distribution and compensate for TMB measurement errors, joint *p*-values can characterize patients’ clinical performances with a single metric. Meanwhile, the *p*-value is the only CFDA-approved metric representative of statistical significance with good interpretability and is acceptable to clinicians. An optimization target of minimizing the *p*-value can ultimately produce a significant TMB classification that distinguishes ICI therapeutic advantages.

#### 2.1.1 Mixed-endpoint joint probability considering TMB errors

Given *n* patients, for patient *i* (*i*=1,…,*n*), *R_i_* represents the status of tumor response (*R_i_* =1,0 for complete response (CR) and partial response (PR), stable disease(SD) and progressive disease (PD), respectively) and *T_i_* denotes the observed event time, which is the minimum of the true event time $T_{i}^{*}$ and the censoring time *C_i_*, that is, $T_{i\;}=\text{min}\left(T_{i}^{*},C_{i}\right)$ . $\delta_{i}=I\left(T_{i}^{*}\leq C_{i}\right)$ defines the event indicator, where *I*(·) is the indicator function. To comprehensively characterize the therapeutic advantages of ICI for patients based on the recorded data, we merged the ORR and TTE endpoints to profile each patient’s prognosis.

For ORR endpoint, the probability of favorable tumor response for patient *i* is expressed as Pr(*R_i_* = 1|*TMB_i_*). For TTE endpoint, the survival probability up to time *t* for patient *i* is $\text{Pr}\left(T_{i}^{*}>t|TMB_{i}\right)=S_{i}(t)$ , where *S_i_* (*t*) denotes the survival function. Due to some shared unobserved features, different endpoints may be intimately connected in practice as they all come from the same patient. Including multiple endpoints in the analysis can, first, increase the power of statistical tests and, second, provide a more comprehensive picture of disease efficacy, for which a single measure does not offer sufficient representation. Therefore, the joint probability incorporating ORR and TTE endpoints is preferable for the comprehensive efficacy assessment for patients undergoing immunotherapy.

The derivation of joint probability $\text{Pr}\left(R_{i}=1,\;T_{i}^{*}>T_{0}|TMB_{i}\right)$ entails examining the correlation structure between various clinical outcomes; indeed, ignoring such an association can lead to higher type I and type II errors (32). The underlying dependency between tumor response and the survival process is commonly illustrated by the introduction of random effects. This study proposes a joint statistics model with increased generality in correlation capture, and *via* a generalized linear mixed model (GLMM) formulation for the efficient estimation of model parameters. We formed a multinomial logistic regression to engage with multicategorical tumor response and a Cox proportional hazard regression for the survival process. The random effect *u* on the ORR endpoint and random effect *v* on the TTE endpoint are set to account for intra-subject correlation, assumed to follow a multivariate normal distribution. Specifically, we extend the GLMM approach of McGilchrist (33) to facilitate efficient statistical inference.


(1) $$\text{Pr}\left(R_{i}=1,T_{i}^{*}>T_{0};\hat{\theta }\right)=\text{Pr}\left(R_{i}=1|{\hat{u}}_{i};\hat{\theta }\right)\text{Pr}\left(T_{i}^{*}>T_{0}|{\hat{v}}_{i};\hat{\theta }\right)\text{Pr}\left({\hat{u}}_{i},{\hat{v}}_{i};\hat{\theta }\right)$$

where *T*_0_ is a prespecified survival time, $\hat{\theta }$ is the maximum likelihood estimate (MLE) of the joint likelihood, ${\hat{u}}_{i}$ and ${\hat{v}}_{i}$ are the point estimates of random effects on respective endpoints obtained by the empirical Bayes method. Details on joint modeling of ORR and TTE endpoints and the solution of the joint probability is available in Section S1.1–1.2 of the Supplementary Materials; such an approach can bring the statistical alpha level closer to the nominal level and can provide additional information about the relationship.

In addition, the observations of TMB inevitably harbor measurement errors. We hypothesize the observed TMB is subject to the additive measurement error model: $TMB_{i}=TMB_{i}^{*}+e_{i}$ , (*i*=1,…,*n*). The error term *e_i_* is independent and identically normal distributed with mean zero and variance $\sigma_{e}^{2}$ , and is independent of endpoints *R_i_*, *T_i_*, *δ_i_*. Because the true TMB^*^ is not observed, the MLE based on true data cannot be used for joint probability calculation directly from the perspective of inconsistency. To reduce the biasing effect caused by measurement errors and obtain a more robust TMB threshold, we integrated the widely applicable corrected-score with the joint model, resulting in approximately consistent estimators based on the observed data. The corrected ORR&TTE joint probability is as follows:


(2) $$\text{Pr}\left(R_{i}=1,T_{i}^{*}>T_{0};\tilde{\theta }\right)=\text{Pr}\left(R_{i}=1|{\tilde{u}}_{i};\tilde{\theta }\right)\text{Pr}\left(T_{i}^{*}>T_{0}|{\tilde{v}}_{i};\tilde{\theta }\right)\text{Pr}\left({\tilde{u}}_{i},{\tilde{v}}_{i};\tilde{\theta }\right)$$

where $\tilde{\theta }$ , ${\tilde{u}}_{i}$ and ${\tilde{v}}_{i}$ is the approximately consistent estimators under the corrected-joint framework. The complete process is in Section **S1.3** of the Supplementary Materials.

#### 2.1.2 Selection of the optimal thresholds

Given that *k* is the number of thresholds set for categorizing the predictive biomarkers TMB into *k*+1 intervals, let *Cut*_*k*_=(*TMB*_1_, … *TMB*_*k*_) denote the vector of *k* thresholds ordered from smaller to larger. When the number of distinct TMB values within the range of clinical meaningfulness is *m*, all possible combinations of thresholds then have up to $A_{m}^{k}$ kinds, where $A_{m}^{k}$ is the number of permutations of *k* thresholds selected from *m* TMB values. Then, we propose that the vector of *k* thresholds *Cut*_*k*_=(*TMB*_1_, … *TMB*_*k*_) that maximizes the difference in ORR&TTE joint benefit between *k*+1 subgroups of patients is thus the optimal thresholds. Patients are subsequently separated into *k*+1 subgroups based on TMB thresholds, *S*_*j*_={ *R*_*jr*_,*T*_*jr*_,*δ*_*jr*_,*TMB*_*jr*_; *r*=1,…,*n*_*j*_, *j*=1,…,*k*+1 }, where *n_j_* denotes the number of patients in subgroup *j* and Σ*_j_n_j_* =*n*. The joint probability characterizes the positive prognosis of patients with both remission of tumor lesions and prolonged survival time, allowing for a more comprehensive evaluation of the patient’s treatment outcomes. Our optimization objective is the categorization with the minimum *p*-value, which maximizes the difference in the probability of the joint ORR&TTE benefit between subgroups. Thus, given the threshold vector *Cut_k_* and patient subgroups{ *S*_1_, … ,*S*_*k*+1_ }, we measure the joint probability difference *D_k_* between *k*+1 subgroups from the distance metric.


(3) $$\begin{array}{ll} D_{k} & ≜\text{Differences}\;\text{between}\;\left\{S_{1},\ldots ,S_{k+1}\right\}\\ & =\text{Distances}\;\text{between}\;\text{Pr}{\left(R_{r}=1,T_{r}^{*}>T_{0j}|TMB_{r}\right)}_{j},j=1,\ldots ,k+1,r=1,\ldots ,n_{j} \end{array}$$

***Comparison of intergroup discrepancy based on the variance-based distance***. First, we construct a variance-based statistical test to determine the distance between the joint probability means of two or more populations. There are two fundamental explanations for the disparity between the joint probability of various subgroups: i), between-group variations caused by the classification conditions, given as the sum of squares of the deviation between the variable means in each subgroup and the overall mean, given as the sum of squares between-group, *SS_b_*, with the degrees of freedom *df_b_*. ii), individual differences in the joint probabilities of patients, which become within-group differences, denoted as the sum of the squares of the deviations between the variable mean in each subgroup and the variable values in that subgroup, denoted as the sum of squares within-group, *SS_w_*, with intergroup degrees of freedom *df_w_*. Thus, the intergroup distance between joint probabilities is determined by the between-group variance and the within-group variance.


(4) $$\begin{array}{c} D_{k}=\frac{variability\;between\;groups}{variability\;within\;groups}=\frac{SS_{b}/df_{b}}{SS_{w}/df_{w}}\\ =\frac{\sum_{j=1}^{k+1}\left[{\left({\bar{p}}_{j}-\bar{p}\right)}^{2}\times n_{j}\right]/k}{\sum_{j=1}^{k+1}\sum_{r=1}^{n_{j}}{\left(p_{jr}-{\bar{p}}_{j}\right)}^{2}/n-k-1} \end{array}$$

where *p_jr_* denotes the joint ORR&TTE probability for patient *r* in subgroup *j*, ${\bar{p}}_{j}$ denotes the mean joint ORR&TTE probability for subgroup *j*, and $\bar{p}$ denotes the overall mean. When the joint probabilities of the patient population satisfy the following assumptions: independence of records; normality; equality of variances (or “homogeneity”), i.e., the variance of records in groups should be the same, then the statistic *D_k_* follows an F-distribution with *k*, *n* – *k* - 1 degree of freedom. At this point, the *p*-value can be calculated from the *F*(*k*, *n* – *k* – 1) quantile. The test of difference is equivalent to one-way ANOVA.

When the joint probabilities of populations do not fulfill the hypothetical premise of independence, normality, and homogeneity, the nonparametric rank statistic is used to compare more than two populations. The total *n* patients across all *k*+1 groups are ranked based on the calculated joint ORR&TTE probability *p_i_* for *i*th patient. Tied probabilities are allocated the average of ranks they would have received if not tied. The diversity among joint probability subgroups is determined by the between-group rank variance and the within-group rank variance. The rank sum variance between groups should be close to the rank variance of the entire sample. Thus, the test statistic is:


(5) $$\begin{array}{c} D_{k}=\frac{between-group\;rank-sum\;variance}{rank\;variance\;of\;the\;entire\;sample}\\ =\frac{12}{n\left(n+1\right)}\sum_{j=1}^{k+1}\frac{RA_{j}^{2}}{n_{j}}-3\left(n+1\right) \end{array}$$

where *RA_j_* is the rank sum for the *j*th subgroup, $RA_{j}=\sum_{r=1}^{n_{j}}rank\left(p_{jr}\right)$ . When *n* is sufficiently large (the number of observations per subgroup exceeds 5, *n_j_* > 5), *D_k_* follows an approximate χ^2^ distribution with *k* degree of freedom. At this point, the *p*-value can be calculated from the χ^2^(*k*) quantile, and the test of difference is equivalent to the Kruskal-Wallis test.

***Comparison of intergroup discrepancy based on the similarity-matrix-based distance***. In addition, we constructed a nonparametric test to measure the intergroup distance based on the concept of the similarity matrix. The dissimilarity between groups is measured *via* the distance between patients, and then whether the target grouping is meaningful is judged by testing whether the distance between groups is considerably greater than the distance within groups. An *n* × *n* similarity matrix is calculated for the joint probability of *n* patients, where there are various methods for measuring distances, including Euclidean distance, Mahalanobis distance, and Minkowski distance. When the joint probability is one-dimensional, we recommend the standard Euclidean distance. When the study expects to refine the joint probability to be a two-dimensional vector *p_i_* = [*p_Ri_*, *p_Ti_*]^*T*^, we recommend the Mahalanobis distance considering the covariance matrix ***V***:


(6) $$d_{il}=d\left(p_{i},p_{l}\right)=\sqrt{\left(p_{i}-p_{l}\right)\left(V^{-1}\right){\left(p_{i}-p_{l}\right)}^{T}}$$

The yielded similarity matrix is then translated into a rank matrix, and the distance statistic is:


(7) $$\begin{array}{c} D_{k}=\text{between}-\text{group}\;\text{dissimilarity}-\text{within}-\text{group}\;\text{dissimilarity}\\ =\frac{r_{b}-r_{w}}{\frac{1}{4}\left[n\left(n-1\right)\right]} \end{array}$$

where *r_b_* denotes the mean rank of between-group dissimilarities, and *r_w_* denotes the mean rank of within-group dissimilarities. The computational complexity of the *n* × *n* similarity-matrix-based distance is *O*(*n*)^2^.


(8) $$\begin{array}{c} r_{b}=\bar{rank}\left(d_{il}\right),\text{patients}\;i,l\;\text{belong}\;\text{to}\;\text{different}\;\text{subgroups}\\ r_{w}=\bar{rank}\left(d_{il}\right),\text{patients}\;i,l\;\text{belong}\;\text{to}\;\text{the}\;\text{same}\;\text{subgroup} \end{array}$$

As the distance metric does not obey a parametric probability distribution, we obtained the *p*-values by permutation test or boostrapping algorithm.

Then, the optimal threshold vector *Cut_k_* enables significant discrimination of ICI benefits between patient subgroups can be expressed as:


(9) $$Cut_{k}=\left(TMB_{1},\ldots ,TMB_{k}\right)=\underset{k\in A_{m}^{k}}{\text{arg}\;\text{max }}D_{k}$$

To solve eq. (9), TMBcat provides a global assessment of every conceivable way of dividing a patient cohort into *k*+1 TMB level expressions, ultimately using the minimal *p*-value principle to produce the most significant thresholds *Cut_k_*. After selecting the appropriate distance metric statistic *D_k_* based on cancer characteristics, we assessed all possible permutations of *Cut_k_* across a range of clinically meaningful values, with a total of $A_{m}^{k}$ species. Specifically, for each possible form of *Cut_k_*, the differences statistic *D_k_* and the corresponding *p*-value are calculated. We can determine the optimal *Cut_k_* by locating the minimal *p*, namely, the highest *D_k_*-statistic.


(10) $$Cut_{k}=\underset{k\in A_{m}^{k}}{\text{arg}\;\text{min }}\;p-\text{value}\;\text{of}\;D_{k}$$

The TMBcat framework defines the distance statistic *D_k_* as a measure of intergroup discrepancy in the comprehensive prognoses to distinguish immunotherapy patient populations. We provide various calculations of *D_k_* depending on the features of the different carcinomas. Under immunotherapy, different tumors have different clinical manifestations as well as the focus of the therapeutic regimen, where tumor remission and survival prolongation are not equally emphasized in certain cancer types. For example, tumor response is the treatment priority in GI cancers as tumor lesion expansion has a tremendous negative impact on patient survival. However, breast cancer, thyroid carcinoma, and skin cancer, among others, are more likely to result in the prolonged survival of patients. Therefore, when assessing a patient’s ICI treatment outcome, the favorable prognostic probability may be a one-dimensional joint probability *p_i_*, which is applicable to variance-based distance, or it may be in the form of a weighted vector *p*_*i*_=[*ω*_1_ *p*_*Ri*_,*ω*_2_ *p*_*Ti*_]^*T*^, where *D_k_* should be calculated by the similarity-matrix-based distance. At this point, our TMBcat is a general framework suitable for pan-cancer analysis, and the appropriate discrepancy metric statistic can be replaced based on the specific clinical characteristics of the tumor.

#### 2.1.3 Selection of the optimal number of thresholds

We determined the optimal number of TMB thresholds based on intergroup discriminations obtained for *Cut*_*k*=*l*_ and *Cut*_*k*=*l*+1_. The criterion used to assess the need for an additional optimal cut-off point is whether it would enhance the composite intergroup discrimination index. The values of *D*_*k*=*l*_ and *D*_*k*=*l*+1_ across *Cut*_*k*=*l*_ and *Cut*_*k*=*l*+1_ cannot be used directly for comparison because of the non-uniform degrees of freedom. In light of this, we based our judgment on the *p*-value, representing the statistical significance. When the minimal *p*-value may decrease by the inclusion of one patient subgroup, an additional threshold is required:


(11) $$p-\text{value}\;\text{of}\;D_{k=l}<p-\text{value}\;\text{of}\;D_{k=l+1}$$

Finally, a step-by-step tutorial on TMBcat is shown in **Algorithm 1**.

Algorithm 1 Tutorial on TMBcat.
**Data:** observed sample information *S = {R_i_, T_i_, δ_i,_ TMB_i,_ i = 1,…, n}*
**Result:** the optimal TMB categorization number and corresponding thresholds
**1** Jointly modeling the ORR&TTE endpoints for each patient *i*;
**2** Calculate the joint probability pi for each patient *i;*
**3** Give the thresholds number *k* and an optional number of TMB values *m*
**4 for** *any possible permutation ∈ A^k^ _m_
* **do
5**      calculate the inter-group differences *D_k_
*:
**6 if** choosing parametric variance-distance **then
7**           
$D_{k}=\frac{\sum_{j=1}^{k+1}\left[{\left({\bar{p}}_{j}-\bar{p}\right)}^{2}\times n_{j}\right]/k}{\sum_{j=1}^{k+1}\sum_{r=1}^{n_{j}}{\left(p_{jr}-{\bar{p}}_{j}\right)}^{2}/n-k-1}$

**8**            *p-*value obtained by *ANOVA*
**9       end
10     if** *choosing non-parametric variance-distance* **then
11**           
$D_{k}=\frac{12}{n\left(n+1\right)}\sum_{j=1}^{k+1}-3\left(n+1\right)$

**12**           *p-*value obtained by *Kruskal-Wallis*
**13      end
14      if** *choosing non-parametric similarity-matrix-distance* **then
15**           
$D_{k}=\frac{r_{b}-r_{w}}{\frac{1}{4}\left[n\left(n-1\right)\right]}$

**16**           *p*
**-**value obtained by *permutation test*
**17      end
18 end
19** The optimal *Cut_k_
* = arg max *D_k_
* = arg min *p*-value of *D_k_
*;
**20** Give the thresholds number *k* + 1, repeat step 4-19;
/* Judgment of the optimal number of thresholds
**21 if** *p-value of D_k_ < p-value of D_k_
*+1 **then
22**      adding a patient subgroup *k = k* + 1
**23 end
24 return** the optimal TMB categorization number *k* and corresponding thresholds *Cut_k_
*

### 2.2 Cohorts assembly

#### 2.2.1 Experimental cohorts

In this study, 64 patients with R/M NPC who have been treated with anti–PD-(L)1 or anti-CTLA-4 were retrospectively examined. Patients with R/M NPC were consecutively enrolled in two single-arm, phase I trials (NCT02721589 and NCT02593786) between March 2016 and January 2018. In addition, 78 Chinese patients with NSCLC in this study have received anti-PD-(L)1 monotherapy at Sun Yat-sen University Cancer Center between December 2015 and August 2017. The trial designs for the dosage escalation and expansion phases have been discussed before (34–36). Enrollment criteria included: i) aged 18-70; ii) Eastern Cooperative Oncology Group performance status of 0-1; iii) histologically or cytologically confirmed NSCLC or NPC with metastatic disease or locoregional recurrence; iv) failure after at least one prior line of systemic therapy; v) radiologically evaluable. Central nervous system metastases, prior malignancy, autoimmune disease, prior immunotherapy, active tuberculosis infection, pregnancy, or immunosuppressive agent treatment were exclusion criteria. The distribution of patient treatments is shown in **Supplementary Table S1**. Patient characteristics, library preparation, sequencing and bioinformatics procedures are available in Supplementary Materials.

#### 2.2.2 Validation cohorts from public literature

In addition to the above 2 experimental cohorts, we assembled 11 validation cohorts of 943 different patients from publicly available databases and studies, encompassing 453 patients with melanoma (16, 21, 37–39), 407 patients with NSCLC (17, 21, 40, 41), 56 patients with renal cell carcinoma (RCC) (16), and 27 patients with bladder (17) (specific clinical characteristics are shown in **Supplementary Table S2**) as the validation cohorts. Briefly, all of these studies are retrospective studies of immunotherapy, and ICI agents include anti-PD-(L)1, anti-CTLA4, combination anti-CTLA4/anti-PD-(L)1, and only a few other agents. The primary efficacy information we are interested in is ORR assessed by Response Evaluation Criteria in Solid Tumors (RECIST 1.1 (42)) and progression-free survival (PFS) and/or overall survival (OS) outcomes. For TMB calculation, the mutation callings are acquired from the three sequencing platforms. Seven studies perform comprehensive genomic profiling by WES, two of which are called by the standard MC3 pipeline. The other four studies are based on currently available NGS panels for TMB estimation: F1CDx and MSK-IMPACT, which the FDA has approved as practicable diagnostic assays. The sequencing pipeline and diverse TMB thresholds are listed in **Supplementary Table S2**.

## 3 Results

### 3.1 Simulation study for determining TMB thresholds

**Figure 2:**
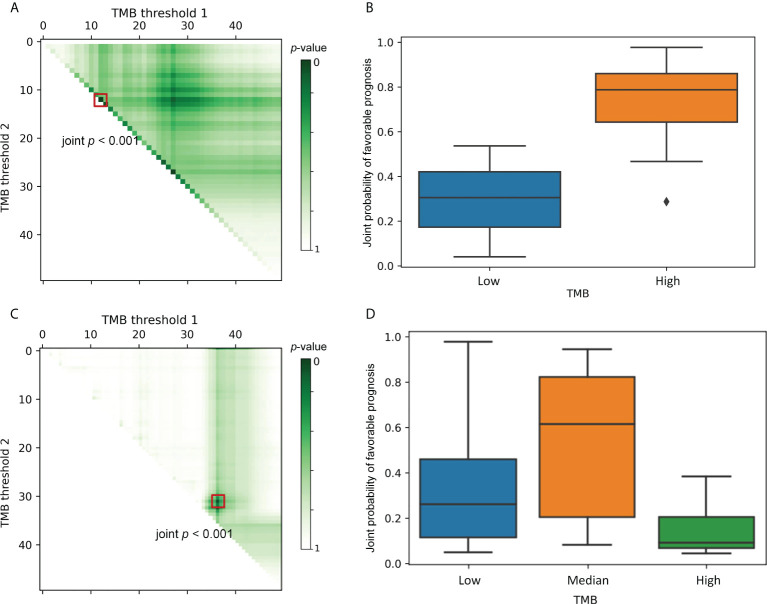
Selection of the optimal thresholds. Each point in left column indicates a particular threshold division. The color intensity represents the joint p-value that depicts the between-group variability of the ORR&TTE joint benefits for patients under that threshold classification. TMB Threshold 1 (on the horizontal axis) and TMB threshold 2 (on the vertical axis) form a categorization dividing the patients into 2–3 different subgroups. The right column shows the comparative prognoses of patients under the optimal TMB categorization corresponding to the left panels. **(A)**, The darkest-colored threshold division point, i.e., the minimum joint *p*-value, appears on the hypotenuse of the right triangle. At this point, k = 1 is the optimal subgroup number, and the boxed point locates the optimal TMB threshold. **(B)**, A comparison of the joint prognostic favorable probability of patients under the optimal TMB classification, clearly indicating that one TMB threshold is sufficient to separate the population into two subgroups with distinct risks. **(C)**, The darkest-colored threshold division point, i.e., the minimum joint *p*-value, appears inside the triangle. The trichotomy is significantly superior to the dichotomy scenario, and the boxed point locates the optimal TMB thresholds. **(D)**, A comparison of the joint prognostic favorable probability of patients under the optimal TMB classification, where a clear stratification effect of the treatment consequences for the three groups of patients can be discerned.

To visualize how our proposed TMBcat determines the optimal TMB thresholds and numbers within a clinically meaningful range, we simulated two classification scenarios of consistent versus inconsistent direction of TMB effects on separate endpoints. Data are simulated in an oncology trial context, with underlying random effects correlated among patients’ ORR and TTE endpoints. The specific modeling process and estimation procedure are in Section **S2** *Simulation* of the Supplementary Materials. Through simulation experiments, we illustrate the applicability of TMBcat for determining TMB categorization. Given clinical practice and computational complexity, the number of patient subgroups is generally compared within 2–5 groups, i.e., *k* = 1-4. The distance metric was tested with the default parametric ANOVA. Owing to the differential direction and magnitude of TMB effects on simulated ORR endpoints versus TTE endpoints, **Figure 2** shows the optimal dichotomous and optimal trichotomous scenarios, respectively.

The data are presented as a right triangular grid, with each point indicating a particular threshold division. The color intensity of each truncated point depicts the between-group variability of the ORR&TTE joint benefits for patients under that threshold classification, with darker colors indicating smaller joint *p*-values. Such a graphical display can shed light on the specific biological basis of the connection between TMB markers and immunotherapy. All probable TMB-high populations are represented on the horizontal axis, with the size becoming smaller from left to right. The vertical axis, which also reflects all possible TMB-low populations, illustrates how their sizes increase as the axis descends. The data along the hypotenuse represents the outcomes of a single threshold that splits the data into two subgroups. Data points away from the hypotenuse up or to the right represent results from two cut-points that define an additional TMB-median population. Greater separation from the hypotenuse results in a larger median subgroup. In **Figure 2A**, the boxed-out darkest-colored threshold division point, i.e., the greatest intergroup distinction, appears on the hypotenuse of the right triangle, where *k* = 1 is the optimal number of classifications. Thus, **Figure 2B** compares patients’ joint prognostic favorable probability under the optimal threshold classification, indicating clearly that one TMB threshold is sufficient to separate the population into two subgroups with different risks. As a comparison, in **Figure 2C**, the darkest-colored point that is boxed out appears inside the triangle, which implies that the joint *p*-value of the optimal TMB tri-classification is significantly smaller than the optimal TMB dichotomous joint *p*-value. The trichotomy is significantly superior to the dichotomy scenario. Similarly, **Figure 2D** compares patient subgroups under the optimal threshold division of the trichotomous categorization, from which we can discern a clear stratification effect of treatment consequences for the three groups of patients. Therefore, in this case, multiple TMB thresholds are supported.

### 3.2 Presence of patients with inconsistent benefiting directions on separate efficacy endpoints

Based on the proposed joint favorable probability, we can yield a comprehensive overview of the response probability and the survival risk of the patient under the mutual modulation represented by the random effects. The joint prognostic indicators can be applied to compare the ICI treatment outcomes simultaneously. For further analysis, we extracted individual patients with inconsistencies between the response indices and survival risk.

**Figure 3:**
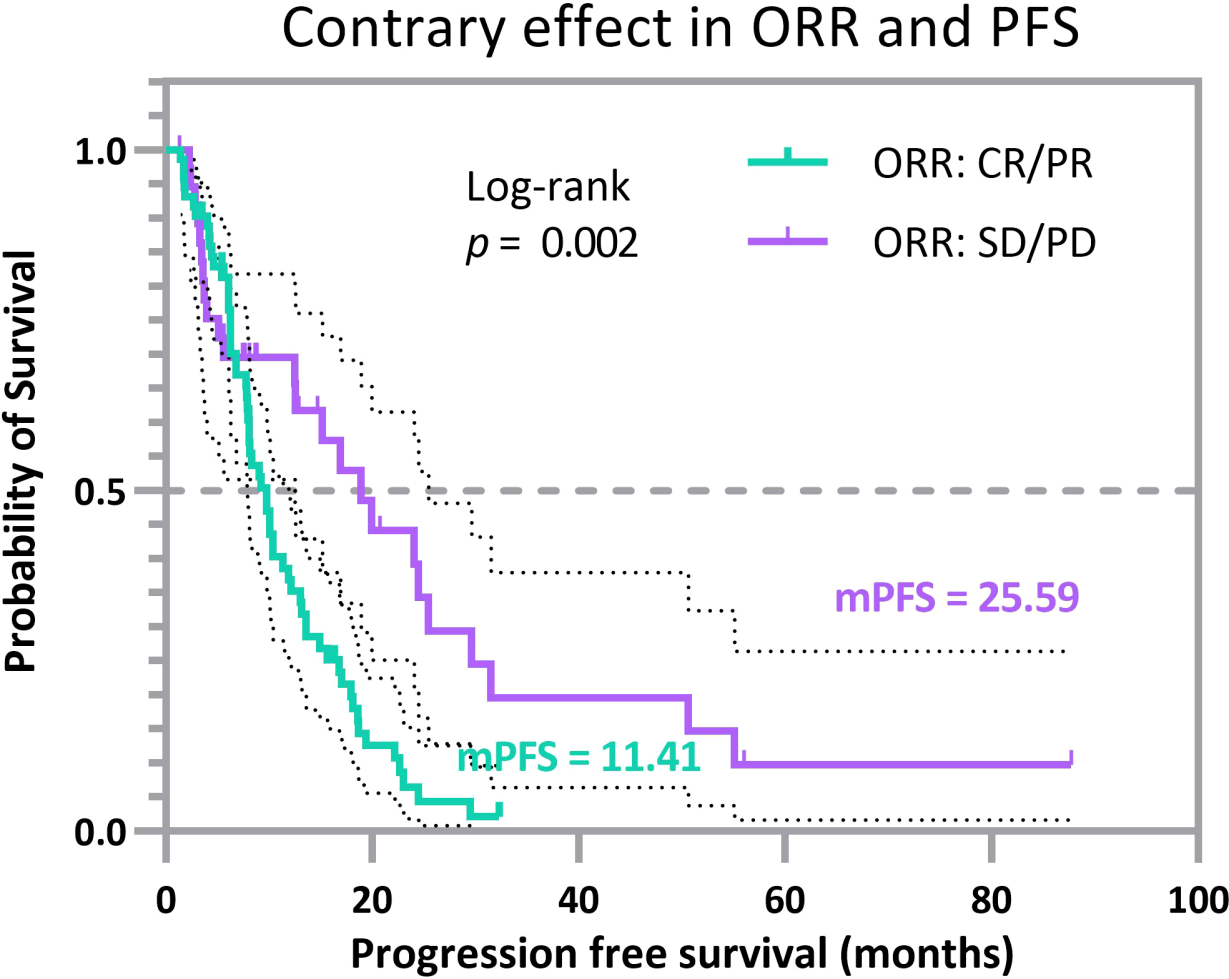
Progression-free survival curves for selected cancer patients with opposite prognosis indices. The lower (green) Kaplan-Meier curve represents patients with CR/PR, but the multi-endpoint joint model directs to SD/PD, and the higher (purple) Kaplan-Meier curve represents patients with SD/PD. Still, the multi-endpoint joint model directs to CR/PR. The clinical benefits of ORR and PFS endpoints point in two distinct directions.

We produced Kaplan-Meier survival curves for PFS to display divergence (**Figure 3**). The lower green curve represents patients with a tumor status of CR/PR, whereas our compound index shows probabilistically that such a trend should not occur in this subgroup. On the opposite, the higher purple curve represents patients with a tumor status of SD/PD, whereas our joint index shows probabilistically that this group tends to possess favorable clinical outcomes. The average PFS of patients in the CR/PR subgroup is 11.409 months (CI, 9.599–13.218 months), and the mPFS of patients in the CR/PR subgroup is 9.8 months (CI, 7.741–11.859 months). In contrast, the average PFS of patients in the SD/PD subgroup is 25.589 months (CI, 15.744–35.435 months), and the mPFS of patients in the SD/PD subgroup is 18.9 months (CI, 12.115–25.685 months). The log-rank test measures the difference between two survival curves, with a significant *p*-value of 0.002. These results identify some clinically overlooked populations: a cohort of patients that tended to survive with tumors, i.e., the group of patients demonstrated in the purple curve (**Figure 3**), revealing an apparently prolonged PFS even though endowed with relatively poorer outcomes in terms of response rubrics. In addition, a cohort of patients whose tumors have resolved may experience rapid disease progression within the first year of treatment, i.e., the group of patients demonstrated in the green curve (**Figure 3**). These patients are from the 2 experimental sets and 11 validation sets, representing a total of 110 individuals accounting for over 10% of the surveyed cohorts. Thus, we offer a bold and novel conclusion: a subset of patients whose effects in two different efficacy endpoints may be of different magnitudes or even point in different directions. This suggests the necessity of our proposal that multiple classifications of TMB should be performed.

Such divergent results reflect, to some extent, the reasonableness of the proposed joint probability in providing a more comprehensive picture of disease efficacy expressed in multifaceted forms when a single endpoint cannot fully represent the complexity of a disease. This issue also reflects that the populations represented by the two curves in **Figure 3** are not specific individual cases, but a small cohort that will negatively impact the whole analysis and even the stratification of patients and should receive more attention in clinical analysis.

### 3.3 Triple classification of patients on TMB level appears more reasonable

Owing to the presence of a subset of patients whose clinical benefits are opposite at two endpoints, further refinement of patient classification based on joint efficacy analysis is warranted. Our clinical cohorts NPC (Panel) and NSCLC were trichotomized by TMBcat, and the analysis of patient grouping results is summarized below.

**Figure 4:**
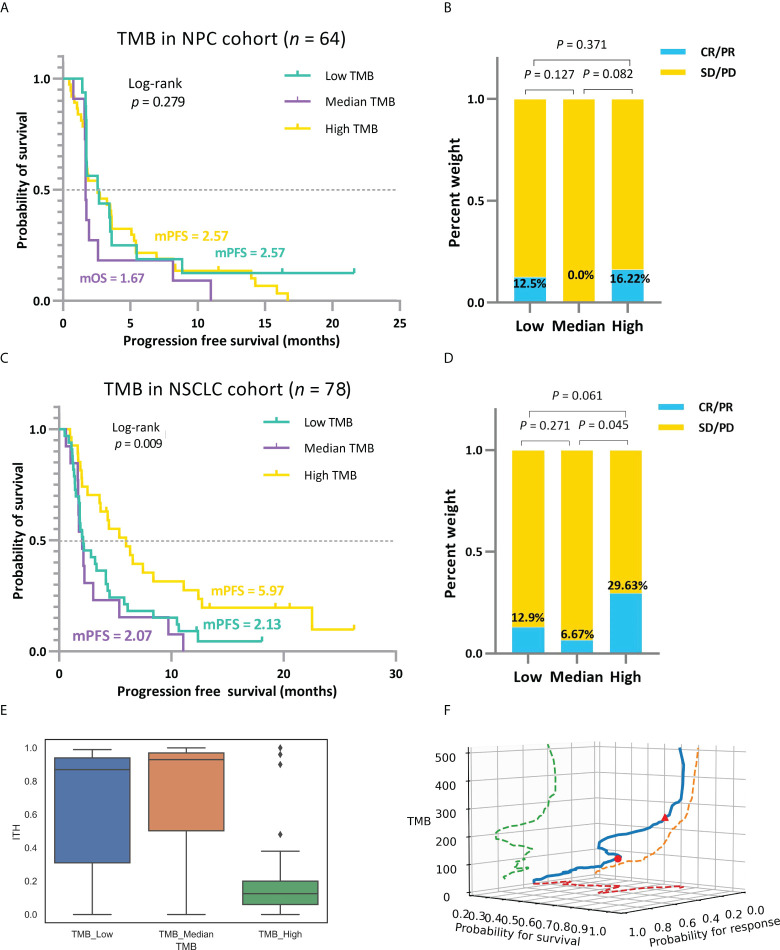
**(A, B)** Based on the mixed-endpoint analysis model, survival curves and ORR comparison for patients with NPC in the low, intermediate, and high TMB groups. **(C, D)** Based on the mixed-endpoint analysis model, survival curves and ORR comparison for patients with NSCLC in the low, intermediate, and high TMB groups. Patients**’** improvements in survival time and response status do not increase strictly linearly with higher TMB values in the scenarios of the multi-classification. Instead, there is a trend of a minor decline followed by a considerable increase in the positive connection between TMB and treatment outcomes. **(E)**, ITH comparison among patients with NSCLC in the low, intermediate, and high TMB groups. **(F)**, Three-dimensional spatial diagram of the association between TMB markers and ICI benefit.

**Figure 4** unfolds the hierarchical results formed by analyzing two different cancer datasets utilizing the TMBcat model, performing Kaplan-Meier survival analyses for TTE and Mann-Whitney U tests for the ORR. We found that an improvement in patient’s survival time did not increase linearly with higher TMB values in the scenarios of the multi-classification. Patients in the *TMB_Median* group confer a poorer prognosis in both PFS and OS survival curves than in the other two *TMB_Low* and *TMB_High* groups. Patients with advanced NSCLC and NPC with low TMB might derive benefit from immunotherapy. Specifically, the mPFS of patients in the *TMB_Median* group is 1.67 and 2.07 months, respectively, in cases NPC and NSCLC, maintaining the lowest in the respective triple classification, while patients with NPC and NSCLC in the *TMB_Low* group have an mPFS of 2.57 and 2.13 months, and those in the *TMB_High* group have an mPFS of 2.57 and 5.97 months, respectively. Likewise, regarding the objective response, *TMB_Median* groups remain the worst performers, with the lowest ORR of 0.0% and 7.69%, respectively, whereas the *TMB_High* groups retained the highest ORRs of 16.22% and 29.63%, respectively. To interpret the origins of such non-linear trends, we considered another factor influencing tumor resistance: intra-tumoral heterogeneity (ITH). ITH is defined as a spatially or temporally uneven distribution of genomic diversification in an individual tumor (43): this is associated with a poor prognosis in solid tumors (44). Patients with low ITH may perform better in the presentation and recognition of neoantigens during immunotherapy (45). The ITH level for each patient with NSCLC was calculated, and the favorable response to immune agents in the *TMB_Low* subgroup could be partially explained by the lower level of ITH (**Figure 4E** and **Supplementary Table S1**). In addition, for the joint probability distribution in space (**Figure 4F**), we show that the smoothed distribution curve remains with multiple inflection points, which demonstrates the plausibility of our proposed multiple classifications of TMB.

**Figure 5:**
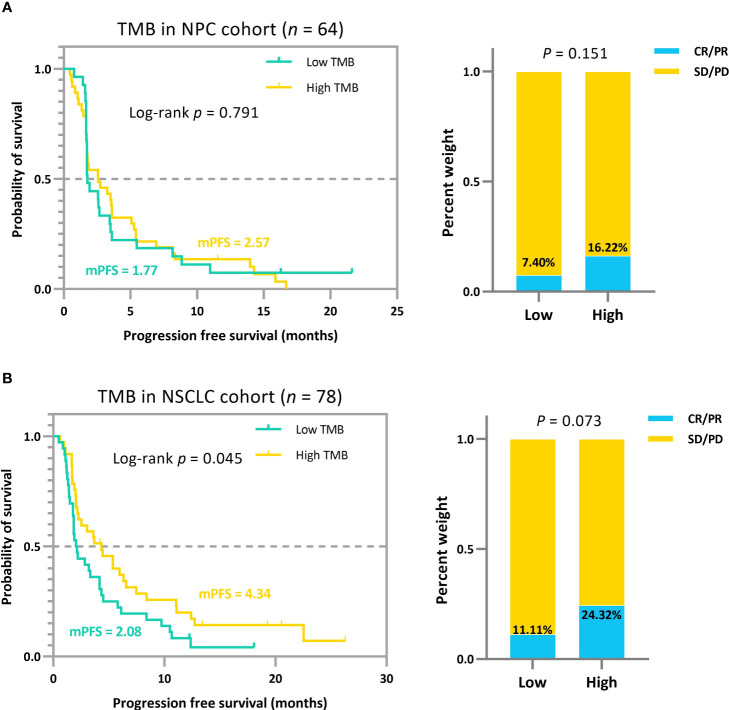
**(A)** Survival curves and ORR comparison for patients with NPC in the low and high TMB groups based on the median. **(B)** Survival curves and ORR comparison for patients with NSCLC in the low and high TMB groups based on the median. The quantile-based TMB subgrouping approach, compared to the minimum joint *p*-value criterion, failed to stratify patient efficacy significantly.

As a comparison, we grouped the clinical cohort NPC (Panel) and NSCLC based on the median TMB, a frequently-used quantile in retrospective analyses (20, 40, 41), and the comparative results of patient efficacy after stratification are shown in **Figure 5**. As TMBcat is optimized with a minimal joint *p*-value, the optimal thresholds for TMB categorization based on our proposed criterion are definitely with the smallest joint *p*-value among all possible threshold divisions. The joint *p*-values for both NPC (Panel) and NSCLC in **Figure 4** are < 0.001, whereas the joint *p*-values for the two cohorts based on the TMB medians in **Figure 5** are 0.521 and 0.061, respectively. To more objectively illustrate the advantages of TMBcat in differentiating patients, we observed the prognoses of patients under the TMB categorization from a single dimension of clinical performance. The differentiation between patient subgroups with the quantile-based TMB categorization is insignificant compared with the proposed minimum joint *p*-value criterion. Both the log-rank *p*-values and Mann-Whitney U *p*-values increased markedly.

In summary, when the efficacy information on two endpoints reveals a consistent direction of benefit, i.e., patients with a higher probability of tumor response tend to have a more extended survival period, which is sufficient to dichotomize patients based on either endpoint. However, when patients display inconsistent benefits on both efficacy endpoints, we propose that it is more reasonable to triclassify patients based on TMB levels in clinical practice, which will help oncologists to screen for patients suitable for immunotherapy.

### 3.4 The TMB subgrouping landscape varies across pan-cancer

The potential association of TMB with sensitivity to ICIs may not be perfectly linear. We performed a pan-cancer analysis for nearly 1,000 patients with cancer in the validation group comprising four cancer types. We identified some novel correlation patterns regarding TMB metrics and immunotherapy efficacy: patients’ clinical improvement did not increase uniformly and linearly with higher TMB values in the multiclassification scenarios.

**Figure 6:**
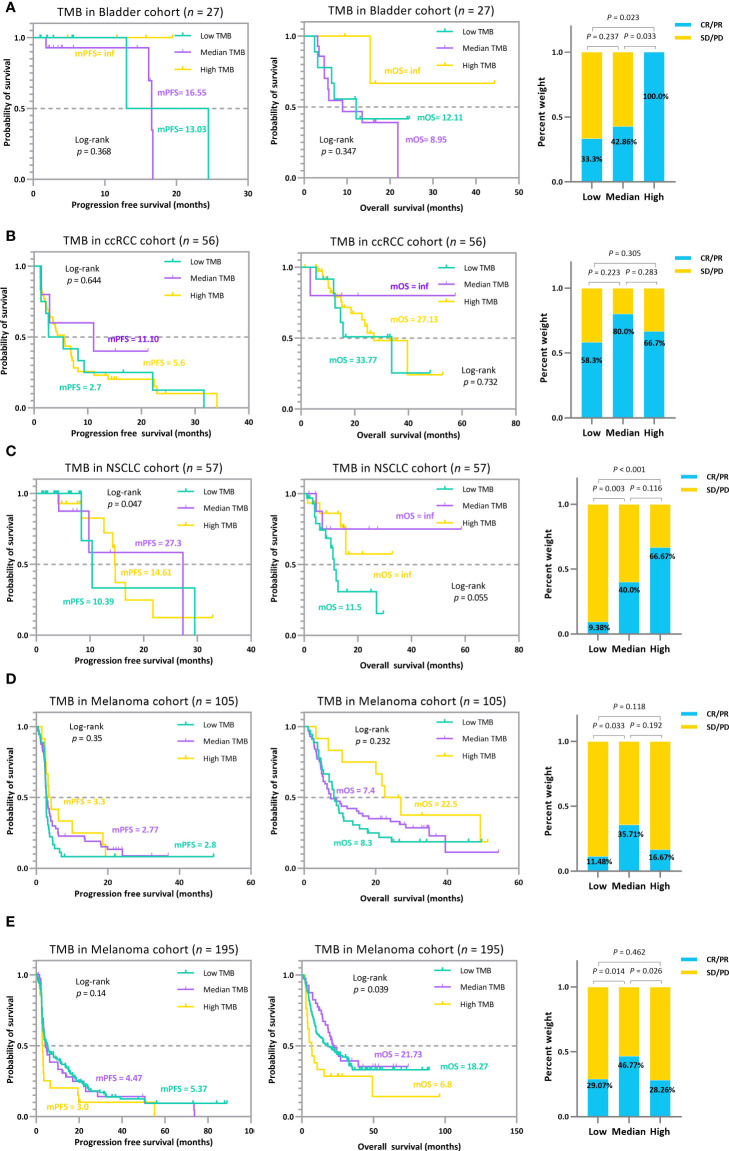
The TMB subgrouping landscape analysis for various cancer types. **(A)**, Kaplan-Meier survival analysis and ORR efficacy comparison for the Bladder cohort. **(B)**, Kaplan-Meier survival analysis and ORR efficacy comparison for the RCC cohort. **(C)**, Kaplan-Meier survival analysis and ORR efficacy comparison for the NSCLC 57 cohort. **(D)**, Kaplan-Meier survival analysis and ORR efficacy comparison for the MEL 105 cohort. **(E)**, Kaplan-Meier survival analysis and ORR efficacy comparison for the MEL 195 cohort. The trichotomy results indicate that the association between TMB index and ICI efficacy is not perfectly linear, i.e., treatment gains do not inherently increase with higher TMB, and the pattern varied across carcinomas.

**Figure 7:**
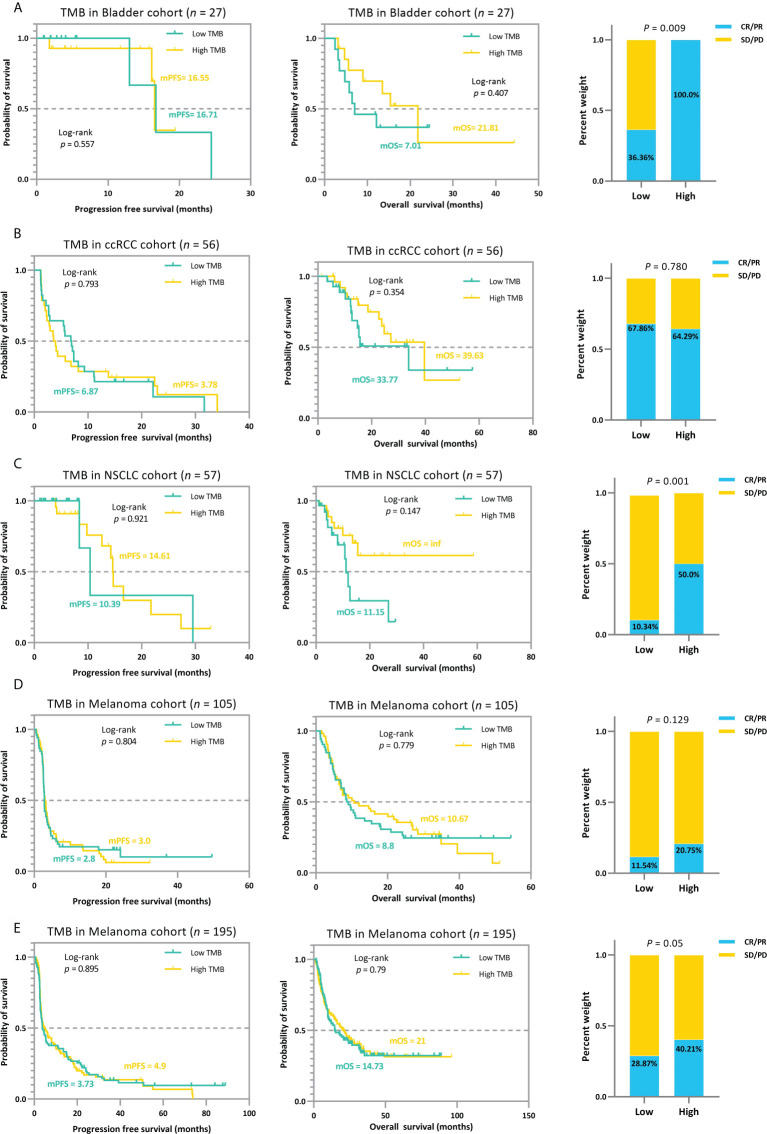
The median-based TMB subgrouping landscape analysis for various cancer types. **(A)**, Kaplan-Meier survival analysis and ORR efficacy comparison for the Bladder cohort. **(B)**, Kaplan-Meier survival analysis and ORR efficacy comparison for the RCC cohort. **(C)**, Kaplan-Meier survival analysis and ORR efficacy comparison for the NSCLC 57 cohort. **(D)**, Kaplan-Meier survival analysis and ORR efficacy comparison for the MEL 105 cohort. **(E)**, Kaplan-Meier survival analysis and ORR efficacy comparison for the MEL 195 cohort. The TMB median cannot distinguish patients’ ICI prognosis and is significantly weaker than the proposed minimum joint *p*-value criterion in terms of statistical significance.

The trichotomy results emphasized that the association between TMB and ICI efficacy is non-linear (**Figure 6**). Patients with RCC, NSCLC, and melanoma in the *TMB_Median* groups display a better trend in ICI outcomes than those in *TMB_Low* and *TMB_High* groups (**Figures 6B–D**). The advantage of the *TMB_Median* groups in terms of survival time is most evident in cases RCC and NSCLC_57, where patients maintain the highest mPFS of 11.1 and 27.3 months (mPFS: 2.7 and 5.6 months for *TMB_Low* and *TMB_High* in case RCC, respectively; log-rank *p*=0.644; mPFS: 10.39 and 14.61 months for *TMB_Low* and *TMB_High* in case NSCLC_57, respectively; log-rank *p*=0.047), and the highest median overall survival (mOS) of inf, inf (mOS: 33.77 and 27.13 months for *TMB_Low* and *TMB_High* in RCC, respectively; log-rank *p*=0.732; mOS: 11.5 months and inf for *TMB_Low* and *TMB_High* in NSCLC_57, respectively; log-rank *p*=0.055; **Figures 6B, C**). On the other hand, when evaluating from ORR, *TMB_High* groups acquire the most improvement only in Bladder and NSCLC_57 cases, do the proportions of tumor response gain as the TMB value increases, ranging from 33.3% to 100.0%, and 9.38% to 66.67%, respectively (**Figures 6A, C**). In the other validation cases, ORRs in *TMB_Median* subgroups reach the peak at 80.0%, 35.71%, and 46.77% in the RCC, Melanoma_105, and Melanoma_195 sets, respectively (**Figures 6B, D, E**). The results for the remaining validation cohorts can be found in **Supplementary Figure S2–7**. In addition, similar to the previous subsection, we performed a subgrouping analysis using the TMB medians for the five validation cohorts to allow a comparison with our proposed TMBcat; the results are summarized in **Figure 7**. Quantile-based TMB subgroups were intuitively weaker than TMBcat in *p*-value comparisons, and median TMB did not distinguish the clinical benefits of patients receiving immunotherapy.

**Figure 8:**
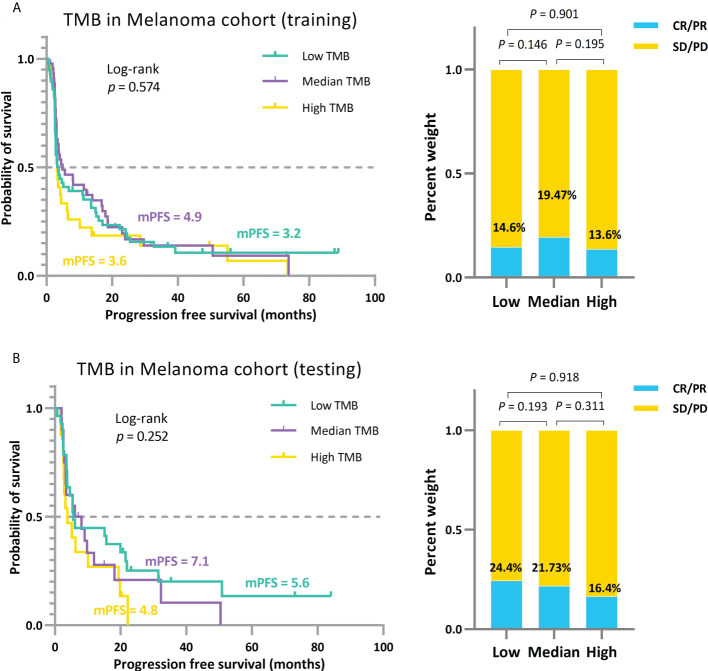
Independent validation of the approach for comprehensively determining the threshold for positive TMB based on TMBcat. **(A)**, The trichotomous treatment effects of patients under the TMB thresholds obtained by training with the 130 patients sampled from the MEL 195 dataset. **(B)**, The triple categorized efficacy comparison for the testing patients under the same TMB thresholds.

To avoid overestimating the performance of our model and the overfitting problem, we further partitioned the MEL_195 queue into training and testing sets. Using the TMBcat-based TMB thresholds selection method, we filtered the appropriate triple classification thresholds based on the training set and grouped the patients for comparison (**Figure 8**). Subsequently, the patients in the independent testing set were classified based on the screened TMB thresholds and the outcomes were analyzed (**Figure 8B**). As summarized by the results, patients’ efficacy had a uniform trend across the three distinct groupings. Thus, our method is generalizable and adaptable to other patient cohorts.

**Figure 9:**
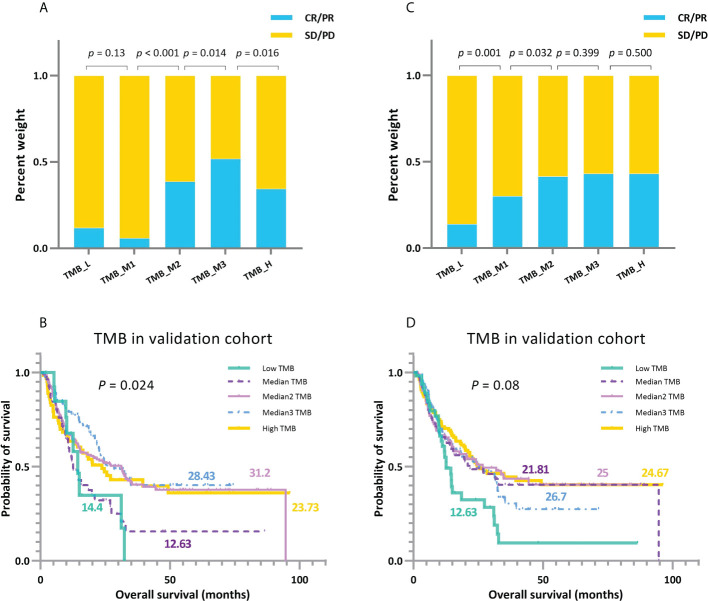
A comparison between TMBcat-based and percentile-based multi-classification. **(A, B)** Grouping results of ORRs and KM survival curves under multi-level division using TMBcat according to TMB levels. **(C, D)** Grouping results of ORRs and KM survival curves under TMB quintiles (cut-offs at 20%, 40%, 60%, and 80%, respectively). The *p*-values in the figures are based on the Mann-Whitney U test and log-rank test, respectively.

To further elaborate this non-linear distribution uniformly, after filtering the panel-based cases, we assembled eight validation clusters for analysis to obtain the multi-classification profiles (**Figure 9**). When patients have extremely high levels of TMB, the effectiveness of immunotherapy is, at this stage, lessened. We speculate that this phenomenon may be due to the accumulation of many mutations in *TMB_High* patients over a long period of carcinogenesis, resulting in heavily differentiated tumors, leading to correspondingly high heterogeneity. At this time, the neo-antigenic activity brought about by high TMB is weakened by the resistance to anticancer therapy brought about by heterogeneity. In contrast, patients with relatively low TMB may be in the early stages of carcinogenesis and have not yet accumulated a sufficient number of mutations; thus, they may gain a small improvement from ICI. Per this non-linear feature, an inverted U-shaped association between patients’ TMB levels and ICI benefits can be clearly observed in melanoma and RCC (**Figures 6B, D, E**, **Supplementary Figures S2, S4**), i.e., poorer performance in patients with high TMB. In contrast, tumors of the skin and kidney typically exhibited a high degree of tumor heterogeneity. In lung cancers with low numbers of tumor clones, this correlation becomes U-shaped or linear, i.e., *TMB_Low* patients may possess better outcomes (**Figure 6C**, **Supplementary Figures S5–7**). This observation also coincides with the relationship between ITH and tumor resistance (44). Similarly, the comparison between the left and right columns (**Figures 9**) also reflects the superior grouping ability of the TMBcat (*p*-value: <0.001–0.13), whereas the quintile-based grouping neither portrays a non-linear distribution, and the *p*-value does not indicate significance (0.001–0.5).

The results show that the association between TMB and ICI efficacy does not present a strict linear increasing trend but instead a non-linear distribution in which low TMB does not preclude response and high TMB is not a sufficient predictor. As seen from the pan-cancer results, multiple thresholds were prevalent, and the thresholds across carcinomas and protocols varied. Our multi-endpoint model provides an integrated and general approach for clinical threshold delineation. The reasons for this non-linear distribution and the underlying driving mechanism are still unclear; further exploratory clinical trials are needed.

## 4 Discussion

Tumor mutation burden has recently become an area of interest; high TMB is associated with a better response to ICI therapies. However, the threshold defining the TMB-high/TMB-positive patients in clinical practice is controversial, and this is exacerbated by the presence of multiple evaluation metrics and TMB inaccuracy. The existing approaches to identify the TMB threshold are merely based on a single endpoint, which may yield excessive information loss to provide statistically significant stratification results. Herein, we describe our solution for TMB threshold selection using a novel criterion named TMBcat, a generalized framework for optimally determining the TMB categorization number and thresholds based on a joint *p*-value. The proposed TMBcat has good scalability because it allows the modeling of the joint distribution and integrates the multidimensional clinical information of patients into a one-dimensional statistic—joint *p*-value, without considering the number of clinical endpoints. In practical applications, when assessing the grouping effect of all possible combinations of TMB thresholds, the number of permutations may be huge when the number of required thresholds *k* and the number of alternative TMB values *m* is large. Thus, an exhaustive search is computationally costly. In these circumstances, we reduce the size of the search space by sampling the data with reasonable segmentation and use heuristic search algorithms, such as simulated annealing, to improve computational efficiency.

In addition, our analyses revealed a novel association pattern, in which the positive correlation between TMB and ICI outcomes was non-linear. In terms of overall trends, patients do not strictly derive more clinical benefits as their TMB levels increase; indeed, TMB-low patients are not necessarily inaccessible to immunotherapy, while patients with extremely high TMB do not always experience the greatest improvements from ICI. These phenotypes may be explained by the fact that cancer patients with remarkably high TMB levels generally accumulate many mutations during their long period of carcinogenesis and that their tumors have become highly differentiated, resulting in complex heterogeneity that confers patients with poor prognoses. Moreover, patients with relatively low TMB may expect a little improvement from ICI because they are in the early stages of cancer development, and many mutations have not yet developed. This phenomenon deserves to be explored in further clinical trials aimed at identifying the patients who may genuinely benefit from treatment with ICIs, refining the therapeutic selection and tailoring the treatment strategy.

Collectively, our results shed new light on TMB multi-stratification based on a multi-endpoint joint assessment of immunotherapy benefits, suggesting that clinicians should consider multiple thresholds. Current evidence on the atypical correlation between TMB and ICI outcomes emphasizes further exploring the corresponding immunobiological mechanisms before wider clinical implementation. All data associated with this study are presented in the Supplementary Materials and Tables.

## 5 Conclusion

Given the fusion of cross-scale, multimodal information and scheme decision-making in immunotherapy, clinical data should be integrated to achieve a comprehensive analysis of patient outcomes. Therefore, we proposed a minimal joint *p*-value criterion from the perspective of differentiating the comprehensive therapeutic advantages, termed TMBcat, to optimize TMB categorization across distinct cancer cohorts; this method surpassed known benchmarks. Previous studies have typically derived only one threshold to divide the immunotherapy patient population into two subgroups, which is largely insufficient. Instead, we consider a multi-threshold categorization incorporating multiple clinical endpoints, a first-of-its-kind pan-cancer framework for TMB categorization.

Based on our proposed optimization framework, we performed our multi-endpoint analysis on 78 patients with NSCLC and 64 patients with NPC who underwent ICI treatments, as well as an assembled cohort of 943 patients included in 11 published studies. Our study identified more novel medical findings compared with the available studies. From the results, we reasonably conclude that: i) the TMB metric is closely associated with immunotherapy benefits, although this association is non-linear and varies between cancer types; ii) integrating multi-dimensional information for patients to employ multi-endpoint joint analysis can prompt a more comprehensive TMB subgrouping; iii) patients receiving immunotherapy may have different effects on different efficacy endpoints, which suggests that iv) there is more than one TMB inflection point available that permit significantly different clinical outcomes in subgroups of patients; and finally, v) the ability of our model TMBcat to provide the optimal number of subgroups in addition to the corresponding TMB thresholds may better assist physicians in treatment decision-making.

## Data availability statement

The original contributions presented in the study are included in the article/**Supplementary Material**. Further inquiries can be directed to the corresponding authors.

## Ethics statement

This study was reviewed and approved by Ethical Review Committee of Sun Yat-sen University Cancer Center. The patients/participants provided their written informed consent to participate in this study.

## Author contributions

YW, XL, JW, and WF conceived and designed the study. YW, XL, and JW developed the methodology. WF, YS, LZ, YW, XL, and YX collected and managed the data. YW wrote the first draft. YW, XL, JW, LZ, and WF reviewed, edited, and approved the manuscript. XL, JW, YX, XPZ, XYZ, YL, LZ, and WF provided administrative, technical, or material support. JW was primarily responsible for the final manuscript. All authors contributed to the article and approved the submitted version.

## Funding

This work was funded by the National Natural Science Foundation of China, grant number 92046009; the Natural Science Basic Research Program of Shaanxi, grant number 2020JC-01; the National Natural Science Foundation of China, grant number 82173101; and the National Natural Science Foundation of China, grant number 81972556.

## Acknowledgments

We thank the patients and their families for participation in the study.

## Conflict of interest

Author YS is employed by Nanjing Geneseeq Technology Inc.

The remaining authors declare that the research was conducted in the absence of any commercial or financial relationships that could be construed as a potential conflict of interest.

## Publisher’s note

All claims expressed in this article are solely those of the authors and do not necessarily represent those of their affiliated organizations, or those of the publisher, the editors and the reviewers. Any product that may be evaluated in this article, or claim that may be made by its manufacturer, is not guaranteed or endorsed by the publisher.
