# Supplementary material for: TMBcat: A multi-endpoint p-value criterion on different discrepancy metrics for superiorly inferring tumor mutation burden thresholds
Source: Front Immunol. 2022 Sep 16;13:995180. doi: 10.3389/fimmu.2022.995180 (PMC9523486; doi:10.3389/fimmu.2022.995180)
Supplement: Supplementary file 3 [file DataSheet_1.pdf]

Supplementary Figures

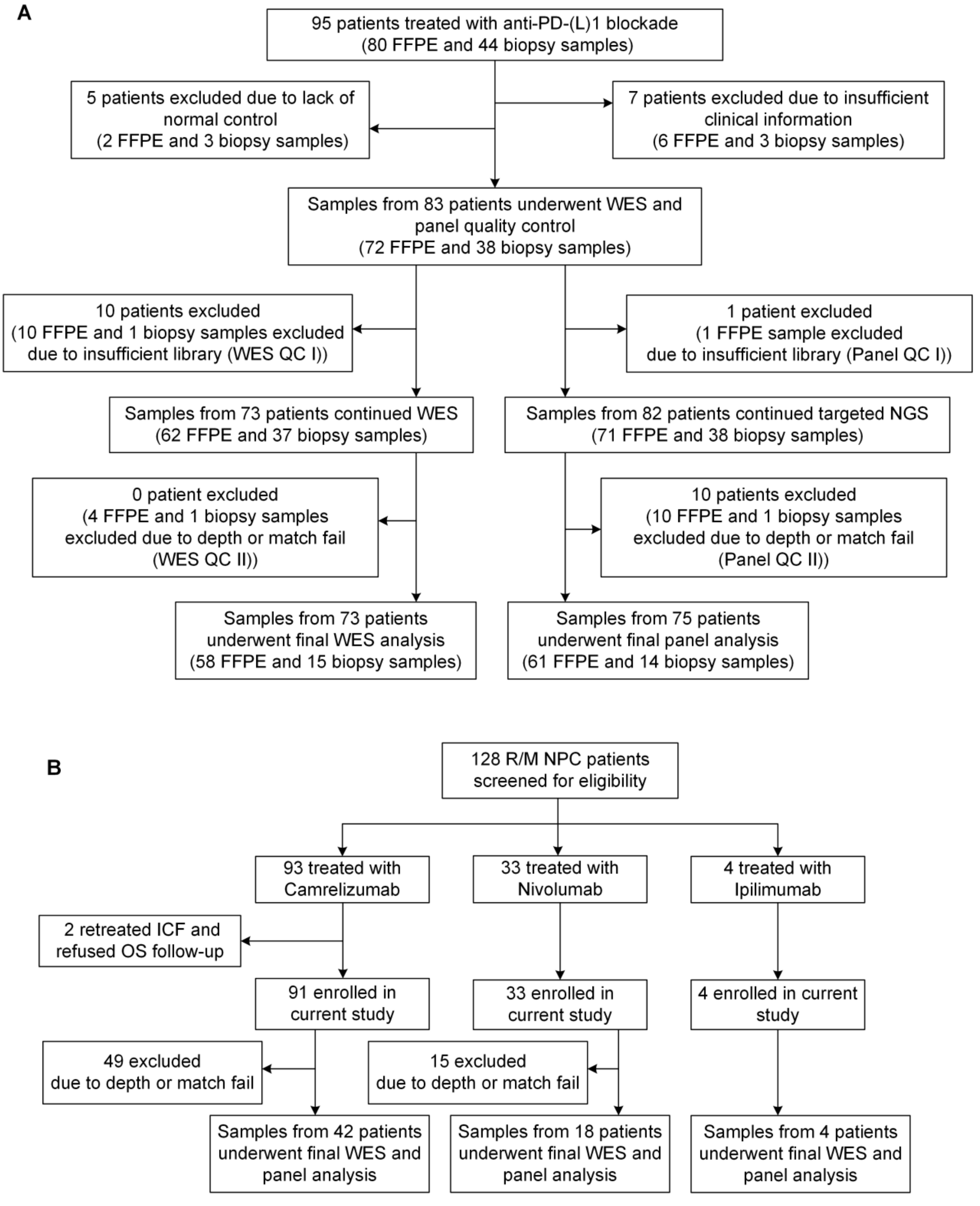

Figure S1 Patient samples included in the final analysis.

A) Flowchart for NSCLC sample inclusions. Among the 95 patients who underwent anti-PD-(L)1 therapies and had available FFPE and/or biopsy tumor samples, we performed WES on samples from 73 patients and targeted NGS on samples from 75 patients. **B**) Flowchart for NPC sample inclusions. Among the 128 patients who underwent anti-PD-(L)1 or anti-CTLA-4 therapies, we performed WES and targeted NGS on samples from 64 patients.

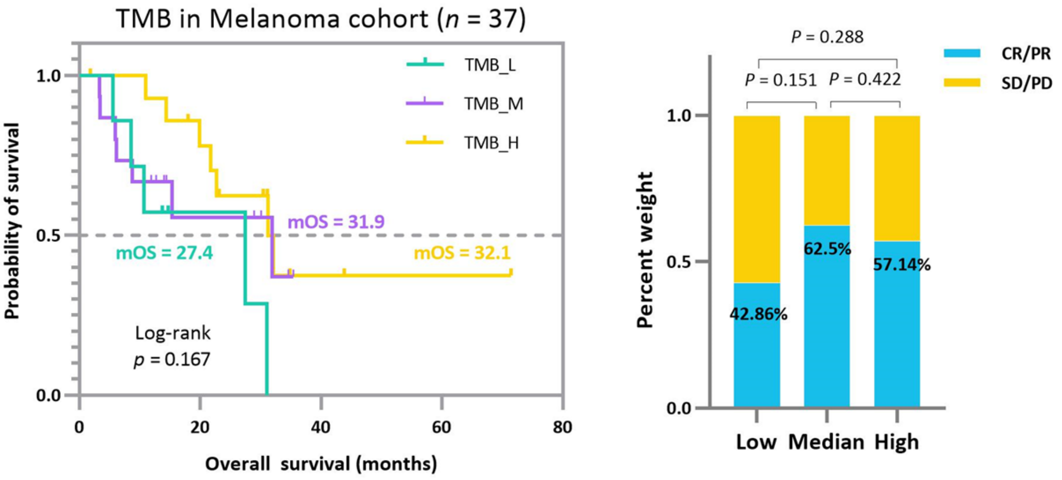

Figure S2 Survival curves and ORR comparison between Melanoma_37 patients.

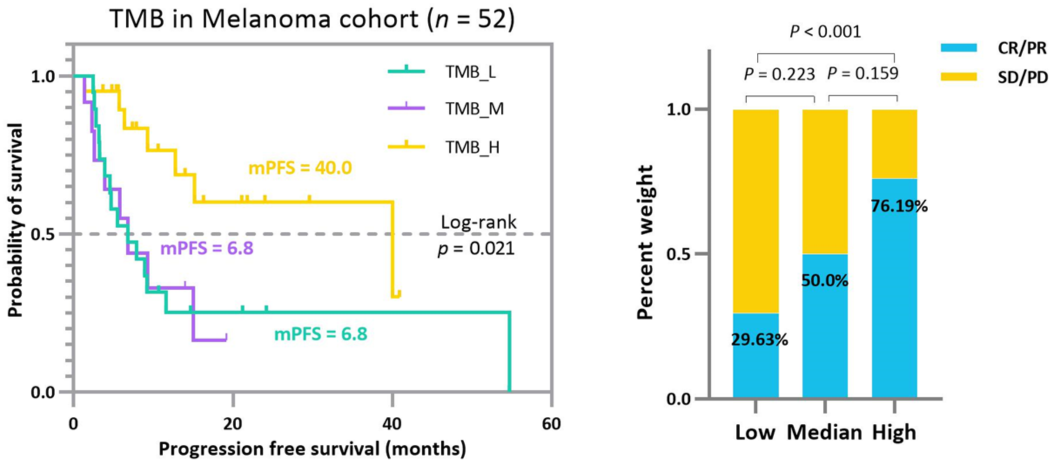

Figure S3 Survival curves and ORR comparison between Melanoma_52 patients.

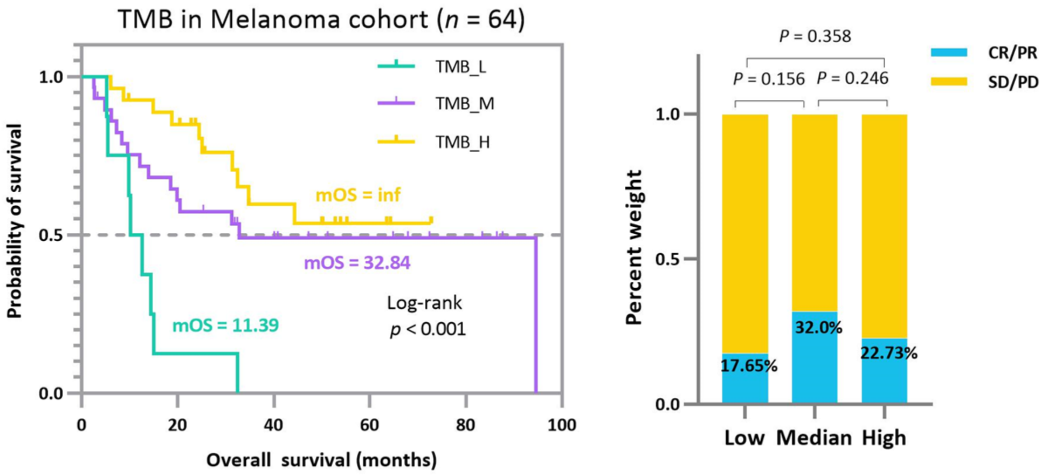

Figure S4 Survival curves and ORR comparison between Melanoma_64 patients.

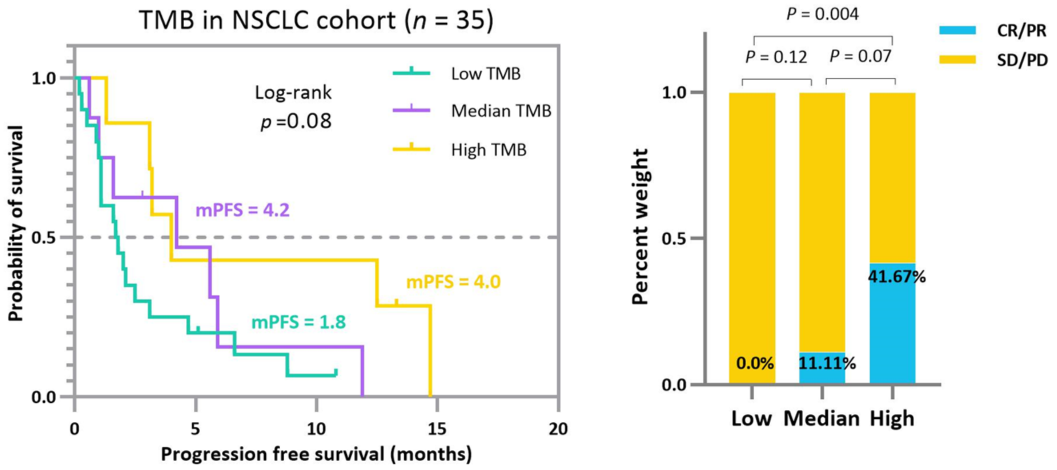

Figure S5 Survival curves and ORR comparison between NSCLC_35 patients.

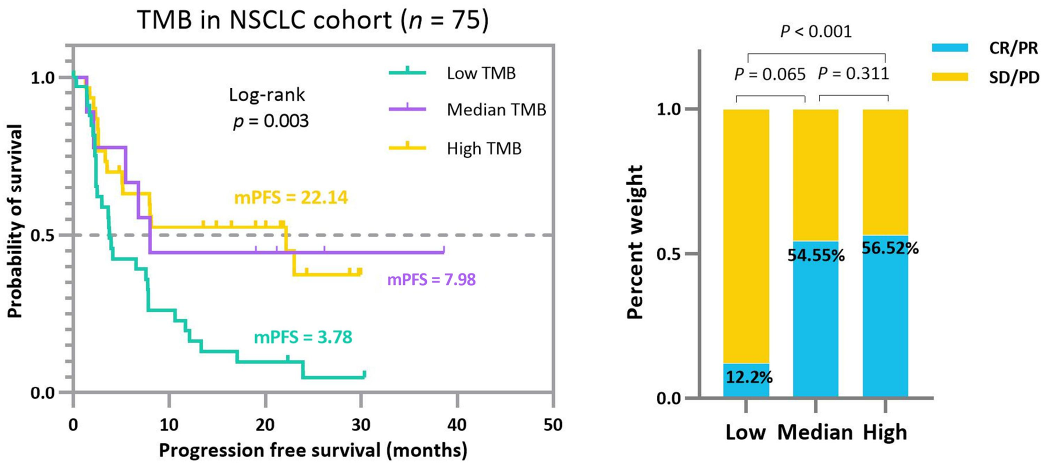

Figure S6 Survival curves and ORR comparison between NSCLC_75 patients.

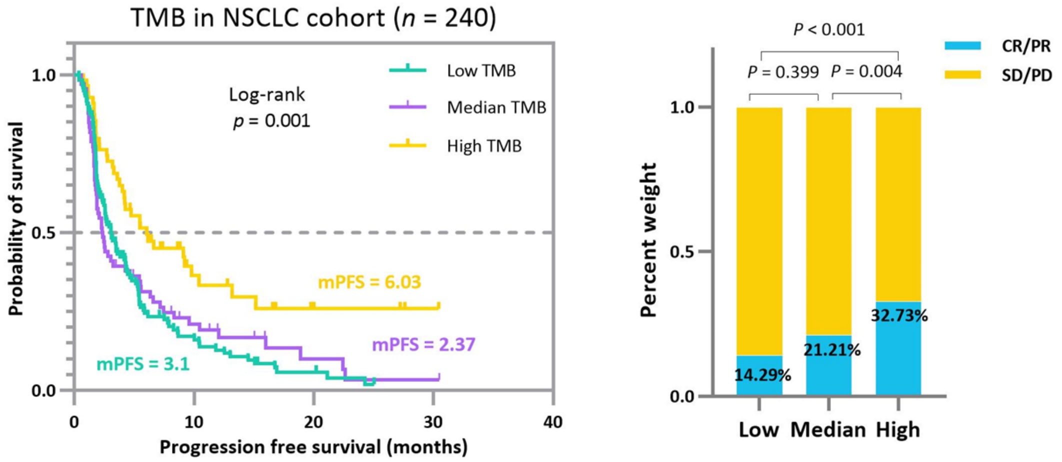

Figure S7 Survival curves and ORR comparison between NSCLC_240 patients.
