# Supplementary material for: TMBcat: A multi-endpoint p-value criterion on different discrepancy metrics for superiorly inferring tumor mutation burden thresholds
Source: Front Immunol. 2022 Sep 16;13:995180. doi: 10.3389/fimmu.2022.995180 (PMC9523486; doi:10.3389/fimmu.2022.995180)
Supplement: Supplementary file 4 [file DataSheet_2.pdf]

Supplementary Material

1 JOINT MODELING OF ORR&TTE ENDPOINTS

1.1 Model specification

Assume the observed data were collected from n patients. We suppose that there are K unique response states for the ORR endpoints. Let R_i denotes the response for patient i . Given the covariates X_i, the conditional probability for R_i is defined as

$$\begin{cases} \pi_k(x_i) = P(R_i = k \mid X_i) = \dfrac{\exp\{g_k(x_i)\}}{1 + \sum_{l=1}^{K} \exp\{g_l(x_i)\}} \\ g_k(x_i) = \ln \dfrac{P(R_i = k \mid X_i)}{P(R_i = 0 \mid X_i)} = x_i^{'} \beta_{Rk} - \gamma_k \\ g_0(x_i) = 0 \end{cases} \tag{S1}$$

where $R_i = 0$ is set to be the anchoring group. Due to the limited number of clinically available CR patients in practice, this paper solely considers the case of $K = 3$, i.e., $R_i = 2, 1, 0$ denoting for CR&PR, SD and PD, respectively.

Let T_i^* denotes the underlying time to event for patient i. For the usual right censoring mechanism, we can observe $T_i = min(T_i^*, C_i)$, where C_i denoting the censoring time. Meanwhile, we define the event indicator as $\delta_i = I(T_i^* \leq C_i)$, where $I(\cdot)$ is the indicator function that takes the value 1 if the event is observed, and 0 otherwise. Given the covariates X_i, the hazard function for the ith patient at time t is defined as

$$\lambda(t; X_i) = \lim_{\Delta t \to 0} \frac{\Pr(t \leq T_i^* < t + \Delta t \mid T_i^* \geq t, X_i)}{\Delta t} \tag{S2}$$

The observations are consist of $D = \{R_i, T_i, \delta_i, X_i\}$, $i = 1, ...n$. The random effects are employed to demonstrate the links between various clinical outcomes. Specifically, we will follow the Generalized Linear Mixed Model (GLMM) framework to introduce random effects into the multinomial logistic regression model and proportional hazards function.

$$\begin{cases} g_1(x_{ij}) = x_i^{'} \beta_{R1} + \gamma_1 + U_{i1} \\ g_2(x_i) = x_i^{'} \beta_{R2} + \gamma_2 + U_{i2} \\ \lambda(t; x_i) = \lambda_0(t) \exp(x_i^{'} \beta_3 + V_i) \end{cases} \tag{S3}$$

where $g_0(x_i) = 0$ denotes the reference group, x_i denotes the vector of covariates, $\beta_k = (\beta_{Rk}^{'}, \gamma_k)$, $k = 1$ or 2, denotes the corresponding logistic regression coefficients for response k, and β_3 denotes the risk factors effects on hazard function. A positive value of coefficients in β_{Rk} implies a higher probability of response k associated with the corresponding covariate, while a positive value of coefficients in β_3 implies a higher risk of failure associated with the risk factor. U_{1i}, U_{2i} and V_i, $i = 1, ..., n$, denote random effects of cluster i on the respective endpoint, which are assumed to follow the multivariate normal distribution $N(0, \Sigma)$ with

$$\Sigma = \begin{pmatrix} \theta_1 & \rho_1\sqrt{\theta_1\theta_2} & \rho_2\sqrt{\theta_1\theta_3} \\ \rho_1\sqrt{\theta_1\theta_2} & \theta_2 & \rho_3\sqrt{\theta_2\theta_3} \\ \rho_2\sqrt{\theta_1\theta_3} & \rho_3\sqrt{\theta_2\theta_3} & \theta_3 \end{pmatrix}$$

Let $\mathbf{u}_1 = (U_{11}, ..., U_{1n})^{'}, \mathbf{u}_2 = (U_{21}, ..., U_{2n})^{'}, \mathbf{v} = (V_1, ..., V_n)^{'}$, and $\mathbf{a} = \left(\mathbf{u}_1^{'}, \mathbf{u}_2^{'}, \mathbf{v}^{'}\right)$. Then the vector $\mathbf{a}$ follows the normal distribution with mean zero and covariance matrix $\Omega = \Sigma \otimes I_n$, where I_n is a $n \times n$ unit matrix, and $\otimes$ denotes the Kronecker product of two matrices. We further define the linear predictors as

$$\begin{aligned} \eta_{1i} &= x_i^{'} \cdot \beta_{R1} + \gamma_1 + U_{i1} \\ \eta_{2i} &= x_i^{'} \cdot \beta_{R2} + \gamma_2 + U_{i2} \\ \eta_{3i} &= x_i^{'} \cdot \beta_3 + V_i \end{aligned}$$

Following the GLMM formulation, the random effects are set to be conditionally fixed, then the best linear unbiased prediction (BLUP) type log-likelihood can be written as the sum of following three terms:

$$\begin{aligned} \ell_R &= \sum_i \left[\sum_{k=1}^{2} R_{ki}\eta_{ki} - \log\left\{1 + \sum_{k=1}^{2} \exp(\eta_{ki})\right\}\right] \\ \ell_S &= \sum_i \log\left[\{\lambda_0(t)\exp\eta_{3i}\}^{\delta_i} \times \exp\{-\mathit{\Lambda}_0(t)\exp\eta_{3i}\}\right] \\ \ell_r &= -\frac{1}{2}\left[\log\left\{(2\pi)^{Kn}|\Omega|\right\} + \mathbf{a}^{'}\Omega^{-1}\mathbf{a}\right] \end{aligned} \tag{S4}$$

where $\lambda_0(t)$ is called *baseline hazard function*, and $\mathit{\Lambda}_0(t) = \int_0^t \lambda_0(s)ds$ denotes the corresponding cumulative baseline hazard. To simplify the estimation procedure, the baseline hazard can be eliminated in the conditional partial likelihood function ℓ_S. Following the marginal likelihood argument, we arrange the n distinct failure times in an increasing order by $t_1 < t_2 < \cdots < t_n$. And the log-likelihood with random effects being conditionally fixed, ℓ_S, can be rewritten as

$$\ell_S = \sum_{i=1}^{n} \log \frac{\exp\eta_{3i}}{\sum_{s\in R(t_i)} \exp\eta_{3s}}$$

where $R(t) = \{j : T_j \geq t\}$ is the risk set at $t-$. So the BLUP type log-likelihood is formulated as

$$\begin{aligned} \ell_f &= \ell_R + \ell_S \\ \ell_{blup} &= \ell_f + \ell_r \end{aligned} \tag{S5}$$

Remark: When the response degrades to a dichotomous indicator (clinically, the tumor status is frequently reduced to response or non-response to simplify the study), the multinomial logistic regression in the proposed model degenerates to a binary logistic regression.

1.2 Estimation procedure

To estimate the corresponding regression coefficient vectors β_1, β_2 and the risk effect β_3, the conditionally fixed random effects $\left(\mathbf{u}_1^{'}, \mathbf{u}_2^{'}, \mathbf{v}^{'}\right)$ and the variance component parameters $(\theta_1, \theta_2, \theta_3, \rho_1, \rho_2, \rho_3)$, the estimation procedure can be performed under GLMM framework. The use of GLMM can avoid the need for intractable high-dimensional integration involved in the marginal likelihood methods or time-consuming Monte Carlo approximations in the E-step for maximum likelihood estimation. The original work of McGilchrist extended the BLUP method for linear mixed models (LMMs) to a GLMM setting (with additional random effect terms in the linear predictor) through the residual maximum likelihood (REML) estimation procedures, which involves an iterative numerical procedure:

[i] For given values of θ_1, θ_2, θ_3 and ρ_1, ρ_2, ρ_3, the estimates of β_1, β_2, β_3, $\mathbf{u}_1$, $\mathbf{u}_2$ and $\mathbf{v}$ can be obtained by maximizing the log-likelihood ℓ_{blup} in eq(S5), which is performed via Newton-Raphson iteration.

$$\begin{bmatrix} \hat{\beta}_1 \\ \hat{\beta}_2 \\ \hat{\beta}_3 \\ \hat{\mathbf{u}}_1 \\ \hat{\mathbf{u}}_2 \\ \hat{\mathbf{v}} \end{bmatrix} = \begin{bmatrix} \beta_{10} \\ \beta_{20} \\ \beta_{30} \\ \mathbf{u}_{10} \\ \mathbf{u}_{20} \\ \mathbf{v}_0 \end{bmatrix} + {I_0}^{-1} \left\{ \begin{bmatrix} X_R^{'} & 0 & 0 \\ 0 & X_R^{'} & 0 \\ 0 & 0 & X^{'} \\ Z^{'} & 0 & 0 \\ 0 & Z^{'} & 0 \\ 0 & 0 & Z^{'} \end{bmatrix} \begin{bmatrix} \partial \ell_f / \partial \eta_1 \\ \partial \ell_f / \partial \eta_2 \\ \partial \ell_f / \partial \eta_3 \end{bmatrix} \right.$$
$$- \frac{1}{\theta_1\theta_2\theta_3\left[1-\rho_1^2-\rho_2^2-\rho_3^2+2\rho_1\rho_2\rho_3\right]} \tag{S6}$$
$$\left. \times \begin{bmatrix} 0 \\ 0 \\ 0 \\ \mathbf{u}_{10}\left(1-\rho_3^2\right)\theta_2\theta_3 - \mathbf{u}_{20}\left(\rho_1-\rho_2\rho_3\right)\theta_3\sqrt{\theta_1\theta_2} - \mathbf{v}_0\left(\rho_2-\rho_1\rho_3\right)\theta_2\sqrt{\theta_1\theta_3} \\ -\mathbf{u}_{10}\left(\rho_1-\rho_2\rho_3\right)\theta_3\sqrt{\theta_1\theta_2} + \mathbf{u}_{20}\left(1-\rho_2^2\right)\theta_1\theta_3 - \mathbf{v}_0\left(\rho_3-\rho_1\rho_2\right)\theta_1\sqrt{\theta_2\theta_3} \\ -\mathbf{u}_{10}\left(\rho_2-\rho_1\rho_3\right)\theta_2\sqrt{\theta_1\theta_3} - \mathbf{u}_{20}\left(\rho_3-\rho_1\rho_2\right)\theta_1\sqrt{\theta_2\theta_3} + \mathbf{v}_0\left(1-\rho_1^2\right)\theta_1\theta_2 \end{bmatrix} \right\}$$

where β_{10}, β_{20}, β_{30}, $\mathbf{u}_{10}$, $\mathbf{u}_{20}$, $\mathbf{v}_0$ are initial values of β_1, β_2, β_3, $\mathbf{u}_1$, $\mathbf{u}_2$, $\mathbf{v}$ respectively, and are replaced by their updated estimates in each iteration, for given values of $\theta_1, \theta_2, \theta_3, \rho_1, \rho_2, \rho_3$. $X_R = (X, \mathbf{1})$ indicates the design matrix with added intercepts $\mathbf{1}$. *I* is the negative second derivative of log-likelihood ℓ_{blup} with respect to β_1, β_2, β_3, $\mathbf{u}_1$, $\mathbf{u}_2$, $\mathbf{v}$. Specifically,

$$I = \begin{bmatrix} X_R^{'} & 0 & 0 \\ 0 & X_R^{'} & 0 \\ 0 & 0 & X^{'} \\ Z^{'} & 0 & 0 \\ 0 & Z^{'} & 0 \\ 0 & 0 & Z^{'} \end{bmatrix} \begin{bmatrix} \frac{-\partial^2 \ell_f}{\partial\eta_1\partial\eta_1^{'}} & \frac{-\partial^2 \ell_f}{\partial\eta_1\partial\eta_2^{'}} & \frac{-\partial^2 \ell_f}{\partial\eta_1\partial\eta_3^{'}} \\ \frac{-\partial^2 \ell_f}{\partial\eta_2\partial\eta_1^{'}} & \frac{-\partial^2 \ell_f}{\partial\eta_2\partial\eta_2^{'}} & \frac{-\partial^2 \ell_f}{\partial\eta_2\partial\eta_3^{'}} \\ \frac{-\partial^2 \ell_f}{\partial\eta_3\partial\eta_1^{'}} & \frac{-\partial^2 \ell_f}{\partial\eta_3\partial\eta_2^{'}} & \frac{-\partial^2 \ell_f}{\partial\eta_3\partial\eta_3^{'}} \end{bmatrix} \begin{bmatrix} X_R^{'} & 0 & 0 & Z^{'} & 0 & 0 \\ 0 & X_R^{'} & 0 & 0 & Z^{'} & 0 \\ 0 & 0 & X^{'} & 0 & 0 & Z^{'} \end{bmatrix}$$
$$+ \frac{1}{\theta_1\theta_2\theta_3\left(1-\rho_1^2-\rho_2^2-\rho_3{}^2+2\rho_1\rho_2\rho_3\right)}$$
$$\times \begin{bmatrix} 0 & 0 & 0 & 0 \\ 0 & \left(1-\rho_3^2\right)\theta_2\theta_3 & -\left(\rho_1-\rho_2\rho_3\right)\theta_3\sqrt{\theta_1\theta_2} & -\left(\rho_2-\rho_1\rho_3\right)\theta_2\sqrt{\theta_1\theta_3} \\ 0 & T & \left(1-\rho_2^2\right)\theta_1\theta_3 & -\left(\rho_3-\rho_1\rho_2\right)\theta_1\sqrt{\theta_2\theta_3} \\ 0 & T & T & \left(1-\rho_1^2\right)\theta_1\theta_2 \end{bmatrix}$$

where the mark T implies that the matrix is symmetric.

[ii] To calculate the asymptotic standard errors for the regression parameter estimates $\hat{\beta}_1$, $\hat{\beta}_2$, $\hat{\beta}_3$, we denote

$$I^{-1} = \begin{pmatrix} T_{11} & T_{12} & T_{13} & T_{14} \\ T_{21} & T_{22} & T_{23} & T_{24} \\ T_{31} & T_{32} & T_{33} & T_{34} \\ T_{41} & T_{42} & T_{43} & T_{44} \end{pmatrix}$$

where the block diagonal matrices I^{-1} can be partitioned conformably into $\beta_1 \mid \beta_2 \mid \beta_3 \mid \mathbf{a}$. The corresponding block diagonal matrices T_{11}, T_{22} and T_{33} can provide the asymptotic variance for $\hat{\beta}_1$, $\hat{\beta}_2$, $\hat{\beta}_3$, respectively. Following the GLMM method, given $\hat{\mathbf{a}} = \left(\hat{\mathbf{u}}_1^{'}, \hat{\mathbf{u}}_2^{'}, \hat{\mathbf{v}}^{'}\right)$ and the block diagonal matrix T_{44}, the REML estimates of $\theta_1, \theta_2, \theta_3, \rho_1, \rho_2, \rho_3$ can be obtained by solving the following nonlinear equations:

$$\begin{cases} \text{tr}\Omega^{-1}\dfrac{\partial\Omega}{\partial\theta_m} + \text{tr}\left(T_{44}+\hat{\mathbf{a}}\hat{\mathbf{a}}^{'}\right)\dfrac{\partial\Omega^{-1}}{\partial\theta_m} = 0 \\ \text{tr}\Omega^{-1}\dfrac{\partial\Omega}{\partial\rho_m} + \text{tr}\left(T_{44}+\hat{\mathbf{a}}\hat{\mathbf{a}}^{'}\right)\dfrac{\partial\Omega^{-1}}{\partial\rho_m} = 0 \end{cases} \tag{S7}$$

where $m = 1, 2, 3$.

[iii] Repeat steps [i] and [ii] until convergence criteria are fulfilled.

As the above procedure involves manipulations of large matrices, the computational complexity and cost of the proposed method are extremely high. For example, update based on eq(S6) in Step [i] appears in every iteration and involves the computation of matrices I and I^{-1}, and the time for calculating the standard errors in Step [ii] is also high. But it is needed only once after the simplified REML estimators have been obtained. Furthermore, predictions of the endpoints can be obtained using the empirical Bayes (EB) method to estimate the point estimates and the variances of the random effects.

Following the developments in McGilchrist (1993), simplified version of the above REML estimators for the variance component parameters and their asymptotic standard errors can be obtained.

$$\eta_k^{'} = (\eta_{k1}, ..., \eta_{kn}), (k = 1, 2)$$

$$w_i = \exp \eta_{3i}, a_i = \delta_i / \sum_{j=i}^{n} w_j,$$

$$b_i = \sum_{j=1}^{i} a_j, d = (\delta_i, ..., \delta_n)$$

$$W = \text{diag}\{w_1, ..., w_n\}, A = \text{diag}\{a_1, ..., a_n\}, B = \text{diag}\{b_1, ..., b_n\}$$

We can rewrite $\frac{\partial l_f}{\partial \eta_k}, -\frac{\partial^2 l_f}{\partial \eta_k \partial \eta_k^{'}}, -\frac{\partial^2 l_f}{\partial \eta_1 \partial \eta_2^{'}}, \frac{\partial l_f}{\partial \eta_3}, -\frac{\partial^2 l_f}{\partial \eta_3 \partial \eta_3^{'}}$ as

$$\frac{\partial l_f}{\partial \eta_k} = \left(R_{k1} - \frac{\exp \eta_{k1}}{1 + \exp \eta_{11} + \exp \eta_{21}}, ..., R_{kn} - \frac{\exp \eta_{kn}}{1 + \exp \eta_{1n} + \exp \eta_{2n}} \right)^{'}$$

where $R_{ki} = I(R_i = k)$, $k = 1, 2$.

$$-\frac{\partial^2 l_f}{\partial \eta_k \partial \eta_k^{'}} = \text{diag}\left\{ \frac{\exp \eta_k + \exp(\eta_1 + \eta_2)}{(1 + \exp \eta_1 + \exp \eta_2)^2} \right\}$$

$$-\frac{\partial^2 l_f}{\partial \eta_1 \partial \eta_2^{'}} = \text{diag}\left\{ -\frac{\exp(\eta_1 + \eta_2)}{(1 + \exp \eta_1 + \exp \eta_2)^2} \right\}$$

$$\frac{\partial l_f}{\partial \eta_3} = d - WMA\mathbf{1}$$

$$-\frac{\partial^2 l_f}{\partial \eta_3 \partial \eta_3^{'}} = WB - WMA^2 M^{'} W$$

where $\mathbf{1}^{'} = (1, ..., 1)$.

Adapting from Lai and Yau (2008), the derivatives for Ω and Ω^{-1}, which are now applied to the multi-endpoint joint model, are given as follows:

$$\Omega^{-1} = \frac{1}{\theta_1\theta_2\theta_3 J} \times \begin{pmatrix} (1-\rho_3^2)\theta_2\theta_3 I_n & -(\rho_1 - \rho_2\rho_3)\theta_3\sqrt{\theta_1\theta_2} I_n & -(\rho_2 - \rho_1\rho_3)\theta_2\sqrt{\theta_1\theta_3} I_n \\ T & (1-\rho_2^2)\theta_1\theta_3 I_n & -(\rho_3 - \rho_1\rho_2)\theta_1\sqrt{\theta_2\theta_3} I_n \\ T & T & (1-\rho_1^2)\theta_1\theta_2 I_n \end{pmatrix}$$

$$\frac{\partial \Omega^{-1}}{\partial \theta_1} = \frac{1}{2\theta_1^2\theta_2\theta_3 J} \times \begin{pmatrix} -2(1-\rho_3^2)\theta_2\theta_3 & (\rho_1 - \rho_2\rho_3)\theta_3\sqrt{\theta_1\theta_2} & (\rho_2 - \rho_1\rho_3)\theta_2\sqrt{\theta_1\theta_3} \\ T & 0 & 0 \\ T & 0 & 0 \end{pmatrix}$$

$$\frac{\partial\Omega^{-1}}{\partial\theta_2}=\frac{1}{2\theta_1\theta_2^2\theta_3 J}\times\begin{pmatrix}0 & (\rho_1-\rho_2\rho_3)\,\theta_3\sqrt{\theta_1\theta_2} & 0\\ T & -2\left(1-\rho_2^2\right)\theta_1\theta_3 & (\rho_3-\rho_1\rho_2)\,\theta_1\sqrt{\theta_2\theta_3}\\ 0 & T & 0\end{pmatrix}$$

$$\frac{\partial\Omega^{-1}}{\partial\theta_3}=\frac{1}{2\theta_1\theta_2\theta_3^2 J}\times\begin{pmatrix}0 & 0 & (\rho_2-\rho_1\rho_3)\,\theta_2\sqrt{\theta_1\theta_3}\\ 0 & 0 & (\rho_3-\rho_1\rho_2)\,\theta_1\sqrt{\theta_2\theta_3}\\ T & T & -2\left(1-\rho_1^2\right)\theta_1\theta_2\end{pmatrix}$$

$$\frac{\partial\Omega^{-1}}{\partial\rho_1}=\frac{1}{\theta_1\theta_2\theta_3 J^2}\times$$
$$\begin{pmatrix}2(\rho_1-\rho_2\rho_3)(1-\rho_3^2)\theta_2\theta_3 & -\left\{J+2(\rho_1-\rho_2\rho_3)^2\right\}\theta_3\sqrt{\theta_1\theta_2} & \left\{\rho_3(1+\rho_1^2+\rho_2^2-\rho_3^2)-2\rho_1\rho_2\right\}\theta_2\sqrt{\theta_1\theta_3}\\ T & 2(\rho_1-\rho_2\rho_3)(1-\rho_2^2)\theta_1\theta_3 & \left\{\rho_2(1+\rho_1^2-\rho_2^2+\rho_3^2)-2\rho_1\rho_3\right\}\theta_1\sqrt{\theta_2\theta_3}\\ T & T & 2\left\{\rho_1(\rho_2^2+\rho_3^2)-\rho_2\rho_3(\rho_1^2+1)\right\}\theta_1\theta_2\end{pmatrix}$$

$$\frac{\partial\Omega^{-1}}{\partial\rho_2}=\frac{1}{\theta_1\theta_2\theta_3 J^2}\times$$
$$\begin{pmatrix}2(\rho_2-\rho_1\rho_3)(1-\rho_3^2)\theta_2\theta_3 & \left\{\rho_3(1+\rho_1^2+\rho_2^2-\rho_3^2)-2\rho_1\rho_2\right\}\theta_3\sqrt{\theta_1\theta_2} & -\left\{J+2\left(\rho_2-\rho_1\rho_3\right)^2\right\}\theta_2\sqrt{\theta_1\theta_3}\\ T & 2\left\{\rho_2(\rho_1^2+\rho_3^2)-\rho_1\rho_3(\rho_2^2+1)\right\}\theta_1\theta_3 & \left\{\rho_1(1-\rho_1^2+\rho_2^2+\rho_3^2)-2\rho_2\rho_3\right\}\theta_1\sqrt{\theta_2\theta_3}\\ T & T & 2(\rho_2-\rho_1\rho_3)(1-\rho_1^2)\theta_1\theta_2\end{pmatrix}$$

$$\frac{\partial\Omega^{-1}}{\partial\rho_3}=\frac{1}{\theta_1\theta_2\theta_3 J^2}\times$$
$$\begin{pmatrix}2\left\{\rho_3(\rho_1^2+\rho_3^2)-\rho_1\rho_3(\rho_3^2+1)\right\}\theta_2\theta_3 & \left\{\rho_2(1+\rho_1^2-\rho_2^2+\rho_3^2)-2\rho_1\rho_3\right\}\theta_3\sqrt{\theta_1\theta_2} & \left\{\rho_1(1-\rho_1^2+\rho_2^2+\rho_3^2)-2\rho_2\rho_3\right\}\theta_2\sqrt{\theta_1\theta_3}\\ T & 2(\rho_3-\rho_1\rho_2)(1-\rho_2^2)\theta_1\theta_3 & -\left\{J+2(\rho_3-\rho_1\rho_2)^2\right\}\theta_1\sqrt{\theta_2\theta_3}\\ T & T & 2(\rho_3-\rho_1\rho_2)(1-\rho_1^2)\theta_1\theta_2\end{pmatrix}$$

where $J = 1-\rho_1^2-\rho_2^2-\rho_3^2+2\rho_1\rho_2\rho_3$. Let $J_1=\begin{pmatrix}I_n & 0 & 0\\ 0 & 0 & 0\\ 0 & 0 & 0\end{pmatrix}$, $J_2=\begin{pmatrix}0 & I_n & 0\\ I_n & 0 & 0\\ 0 & 0 & 0\end{pmatrix}$, $J_3=\begin{pmatrix}0 & 0 & I_n\\ 0 & 0 & 0\\ I_n & 0 & 0\end{pmatrix}$, $J_4=\begin{pmatrix}0 & 0 & 0\\ 0 & I_n & 0\\ 0 & 0 & 0\end{pmatrix}$, $J_5=\begin{pmatrix}0 & 0 & 0\\ 0 & 0 & I_n\\ 0 & I_n & 0\end{pmatrix}$, $J_6=\begin{pmatrix}0 & 0 & 0\\ 0 & 0 & 0\\ 0 & 0 & I_n\end{pmatrix}$, and

$$L_1=\mathrm{tr}J_1\left(T_{44}+\hat{a}\hat{a}^{'}\right)\quad L_2=\mathrm{tr}J_2\left(T_{44}+\hat{a}\hat{a}^{'}\right)/2\quad L_3=\mathrm{tr}J_3\left(T_{44}+\hat{a}\hat{a}^{'}\right)/2$$
$$L_4=\mathrm{tr}J_4\left(T_{44}+\hat{a}\hat{a}^{'}\right)\quad L_5=\mathrm{tr}J_5\left(T_{44}+\hat{a}\hat{a}^{'}\right)/2\quad L_6=\mathrm{tr}J_6\left(T_{44}+\hat{a}\hat{a}^{'}\right)$$

Then the estimating equations for the variance component parameters can be rewritten as:

$$f_1=\frac{n}{\theta_1}+\frac{1}{\theta_1^2\theta_2\theta_3 J}\left\{-(1-\rho_3^2)\theta_2\theta_3 L_1+(\rho_1-\rho_2\rho_3)\theta_3\sqrt{\theta_1\theta_2}L_2+(\rho_2-\rho_1\rho_3)\theta_2\sqrt{\theta_1\theta_3}L_3\right\}\tag{S8}$$

$$f_2=\frac{n}{\theta_2}+\frac{1}{\theta_1\theta_2^2\theta_3 J}\left\{-(1-\rho_2^2)\theta_1\theta_3 L_4+(\rho_1-\rho_2\rho_3)\theta_3\sqrt{\theta_1\theta_2}L_2+(\rho_3-\rho_1\rho_2)\theta_1\sqrt{\theta_2\theta_3}L_5\right\}\tag{S9}$$

$$f_3 = \frac{n}{\theta_3} + \frac{1}{\theta_1\theta_2\theta_3^2 J}\left\{-(1-\rho_1^2)\theta_1\theta_2 L_6 + (\rho_2-\rho_1\rho_3)\theta_2\sqrt{\theta_1\theta_3}L_3 + (\rho_3-\rho_1\rho_2)\theta_1\sqrt{\theta_2\theta_3}L_5\right\} \tag{S10}$$

$$\begin{aligned} f_4 = &-\frac{2(\rho_1-\rho_2\rho_3)}{J}n + \frac{1}{\theta_1\theta_2\theta_3 J^2}\times \\ &\Big[2(\rho_1-\rho_2\rho_3)(1-\rho_3^2)\theta_2\theta_3 L_1 - 2\left\{J + 2\left(\rho_1-\rho_2\rho_3\right)^2\right\}\theta_3\sqrt{\theta_1\theta_2}L_2 \\ &+ 2\left\{\rho_3(1+\rho_1^2+\rho_2^2-\rho_3^2) - 2\rho_1\rho_2\right\}\theta_2\sqrt{\theta_1\theta_3}L_3 \\ &+ 2(\rho_1-\rho_2\rho_3)(1-\rho_2^2)\theta_1\theta_3 L_4 \\ &+ 2\left\{\rho_2(1+\rho_1^2-\rho_2^2+\rho_3^2) - 2\rho_1\rho_3\right\}\theta_1\sqrt{\theta_2\theta_3}L_5 \\ &+ 2\left\{\rho_1(\rho_2^2+\rho_3^2) - \rho_2\rho_3(\rho_1^2+1)\right\}\theta_1\theta_2 L_6\Big] \end{aligned} \tag{S11}$$

$$\begin{aligned} f_5 = &-\frac{2(\rho_2-\rho_1\rho_3)}{J}n + \frac{1}{\theta_1\theta_2\theta_3 J^2}\times \\ &\big[2(\rho_2-\rho_1\rho_3)(1-\rho_3^2)\theta_2\theta_3 L_1 \\ &+ 2\left\{\rho_3(1+\rho_1^2+\rho_2^2-\rho_3^2) - 2\rho_1\rho_2\right\}\theta_3\sqrt{\theta_1\theta_2}L_2 \\ &- 2\left\{J + 2(\rho_2-\rho_1\rho_3)^2\right\}\theta_2\sqrt{\theta_1\theta_3}L_3 \\ &+ 2\left\{\rho_2(\rho_1^2+\rho_3^2) - \rho_1\rho_3(\rho_2^2+1)\right\}\theta_1\theta_3 L_4 \\ &+ 2\left\{\rho_1(1-\rho_1^2+\rho_2^2+\rho_3^2) - 2\rho_2\rho_3\right\}\theta_1\sqrt{\theta_2\theta_3}L_5 \\ &+ 2(\rho_2-\rho_1\rho_3)(1-\rho_1^2)\theta_1\theta_2 L_6\ \big] \end{aligned} \tag{S12}$$

$$\begin{aligned} f_6 = &-\frac{2(\rho_3-\rho_1\rho_2)}{J}n + \frac{1}{\theta_1\theta_2\theta_3 J^2}\times \\ &\big[2\left\{\rho_3(\rho_1^2+\rho_2^2) - \rho_1\rho_2(\rho_3^2+1)\right\}\theta_2\theta_3 L_1 \\ &+ 2\left\{\rho_2(1+\rho_1^2-\rho_2^2+\rho_3^2) - 2\rho_1\rho_3\right\}\theta_3\sqrt{\theta_1\theta_2}L_2 \\ &+ 2\left\{\rho_1(1-\rho_1^2+\rho_2^2+\rho_3^2) - 2\rho_2\rho_3\right\}\theta_2\sqrt{\theta_1\theta_3}L_3 \\ &+ 2(\rho_3-\rho_1\rho_2)(1-\rho_2^2)\theta_1\theta_3 L_4 \\ &- 2\left\{J + 2\left(\rho_3-\rho_1\rho_2\right)^2\right\}\theta_1\sqrt{\theta_2\theta_3}L_5 \\ &+ 2(\rho_3-\rho_1\rho_3)(1-\rho_1^2)\theta_1\theta_2 L_6\big] \end{aligned} \tag{S13}$$

After simplifications, we can obtain the analytical solution of the REML estimations for the variance component parameters

$$\hat{\theta}_1 = L_1/n \tag{S14}$$

$$\hat{\theta}_2 = L_4/n \tag{S15}$$

$$\hat{\theta}_3 = L_6/n \tag{S16}$$

$$\hat{\rho}_1 = L_2/\sqrt{L_1 L_4} \tag{S17}$$

$$\hat{\rho}_2 = L_3/\sqrt{L_1 L_6} \tag{S18}$$

$$\hat{\rho}_3 = L_5/\sqrt{L_4 L_6} \tag{S19}$$

To compute the asymptotic variances for the variance component parameter estimates, let

$$K_1 = T_{44}\frac{\partial\Omega^{-1}}{\partial\theta_1} \quad K_2 = \Omega\frac{\partial\Omega^{-1}}{\partial\theta_1} \quad K_3 = T_{44}\frac{\partial\Omega^{-1}}{\partial\theta_2} \quad K_4 = \Omega\frac{\partial\Omega^{-1}}{\partial\theta_2}$$
$$K_5 = T_{44}\frac{\partial\Omega^{-1}}{\partial\theta_3} \quad K_6 = \Omega\frac{\partial\Omega^{-1}}{\partial\theta_3} \quad K_7 = T_{44}\frac{\partial\Omega^{-1}}{\partial\rho_1} \quad K_8 = \Omega\frac{\partial\Omega^{-1}}{\partial\rho_1}$$
$$K_9 = T_{44}\frac{\partial\Omega^{-1}}{\partial\rho_2} \quad K_{10} = \Omega\frac{\partial\Omega^{-1}}{\partial\rho_2} \quad K_{11} = T_{44}\frac{\partial\Omega^{-1}}{\partial\rho_3} \quad K_{12} = \Omega\frac{\partial\Omega^{-1}}{\partial\rho_3}$$

Then, following the arguments in McGilchrist and Yau (1995), the covariance matrix of the estimators in the variance component is given by

$$\text{var}\begin{pmatrix}\hat{\theta}_1\\ \hat{\theta}_2\\ \hat{\theta}_3\\ \hat{\rho}_1\\ \hat{\rho}_2\\ \hat{\rho}_3\end{pmatrix} = 2\begin{pmatrix} a_{11} & a_{12} & a_{13} & a_{14} & a_{15} & a_{16}\\ \cdot & a_{22} & a_{23} & a_{24} & a_{25} & a_{26}\\ \cdot & \cdot & a_{33} & a_{34} & a_{35} & a_{36}\\ \cdot & \cdot & \cdot & a_{44} & a_{45} & a_{46}\\ \cdot & \cdot & \cdot & \cdot & a_{55} & a_{56}\\ \cdot & \cdot & \cdot & \cdot & \cdot & a_{66}\end{pmatrix}^{-1}$$

$$\begin{aligned}
a_{11} &= \text{tr}\,(K_1 - K_2)^2\\
a_{12} &= \text{tr}\,(K_1K_3 + K_2K_4 - 2K_1K_4)\\
a_{13} &= \text{tr}\,(K_1K_5 + K_2K_6 - 2K_1K_6)\\
a_{14} &= \text{tr}\,(K_1K_7 + K_2K_8 - 2K_1K_8)\\
a_{15} &= \text{tr}\,(K_1K_9 + K_2K_{10} - 2K_1K_{10})\\
a_{16} &= \text{tr}\,(K_1K_{11} + K_2K_{12} - 2K_1K_{12})\\
a_{22} &= \text{tr}\,(K_3 - K_4)^2\\
a_{23} &= \text{tr}\,(K_3K_5 + K_4K_6 - 2K_3K_6)\\
a_{24} &= \text{tr}\,(K_3K_7 + K_4K_8 - 2K_3K_8)\\
a_{25} &= \text{tr}\,(K_3K_9 + K_4K_{10} - 2K_3K_{10})\\
a_{26} &= \text{tr}\,(K_3K_{11} + K_4K_{12} - 2K_3K_{12})\\
a_{33} &= \text{tr}\,(K_5 - K_6)^2\\
a_{34} &= \text{tr}\,(K_5K_7 + K_6K_8 - 2K_5K_8)\\
a_{35} &= \text{tr}\,(K_5K_9 + K_6K_{10} - 2K_5K_{10})\\
a_{36} &= \text{tr}\,(K_5K_{11} + K_6K_{12} - 2K_5K_{12})\\
a_{44} &= \text{tr}\,(K_7 - K_8)^2\\
a_{45} &= \text{tr}\,(K_7K_9 + K_8K_{10} - 2K_7K_{10})\\
a_{46} &= \text{tr}\,(K_7K_{11} + K_8K_{12} - 2K_7K_{12})\\
a_{55} &= \text{tr}\,(K_1 - K_{10})^2\\
a_{56} &= \text{tr}\,(K_9K_{11} + K_{10}K_{12} - 2K_9K_{12})\\
a_{66} &= \text{tr}\,(K_{11} - K_{12})^2
\end{aligned}$$

1.3 Measurement errors

To reducing the biasing effect caused by measurement errors and obtain a more robust TMB threshold, we integrated the widely applicable corrected-score with the joint model, resulting in approximately consistent estimators based on the observed data. The covariate $X_i = [Z_i, TMB_i]^T$, where Z_i denotes a vector of covariates, e.g., age, gender, treatment indicator, cancer stage. $TMB_i = TMB_i^* + e_i$, TMB^* is the true TMB value which cannot be observed. Score function is the first order derivative of the likelihood $\Psi = [\frac{\partial l_f}{\partial\eta_1}, \frac{\partial l_f}{\partial\eta_2}, \frac{\partial l_f}{\partial\eta_3}]$. A corrected score is a function Ψ_c^* of the observed data having the important property that

$$\mathbf{E}\left\{\Psi_c^*\left(R_i, T_i, \delta_i, \mathbf{Z}_i, TMB_i^*; \mathbf{\Theta}\right)|R_i, T_i, \delta_i, \mathbf{Z}_i, TMB_i\right\} = \Psi\left(R_i, T_i, \delta_i, \mathbf{Z}_i, TMB_i; \mathbf{\Theta}\right) \tag{S20}$$

which is conditionally unbiased for the true-data score function according to the property of conditional expectation, $\mathbf{E}\left\{\Psi_c^*\left(R_i, T_i, \delta_i, \mathbf{Z}_i, TMB_i^*; \mathbf{\Theta}\right)\right\} = 0$. The corrected scores provide an approach to reducing bias incurred by covariate measurement error.

Based on the correct-score, $\Psi_c^* = [(\frac{\partial l_f}{\partial \beta_{R1}})_c, (\frac{\partial l_f}{\partial \beta_{R2}})_c, (\frac{\partial l_f}{\partial \beta_T})_c]$, specifically:

$$\eta_{1i} = Z_i^{'} \cdot \beta_{R1z} + \widetilde{TMB}_{j,i} \cdot \beta_{R1m} + U_{i1}$$

$$\eta_{2i} = Z_i^{'} \cdot \beta_{R2z} + \widetilde{TMB}_{j,i} \cdot \beta_{R2m} + U_{i2}$$

$$(\frac{\partial l_f}{\partial \beta_{R1}})_c = (\frac{\partial l_f}{\partial \eta_1})_c \cdot X_i = [J^{-1}\sum_{j=1}^{J}\frac{\partial l_f}{\partial (\eta_1)_c}] \cdot X_i \tag{S21}$$

$$(\frac{\partial l_f}{\partial \beta_{R2}})_c = (\frac{\partial l_f}{\partial \eta_1})_c \cdot X_i = [J^{-1}\sum_{j=1}^{J}\frac{\partial l_f}{\partial (\eta_1)_c}] \cdot X_i$$

where the complex variate $\widetilde{TMB}^*_{j,i} = TMB_i^* + \sqrt{-1}\xi_{j,i}$, and $\xi_{j,i}$ is a normal random vector with zero mean and variance σ_e. Since the logistic function does not satisfy the smoothness condition required by the corrected-score theory, it has no intuitively exact corrected score but obtains an approximation by means of complex variable extrapolation. Additionally,

$$(\frac{\partial l_f}{\partial \beta_T})_c = \sum \delta_i \left[X_i - \frac{\sum_{\{j:T_j \geq T_i\}} X_j \exp\left(X_j^\top \beta_T + V_j\right)}{\sum_{\{j:T_j \geq T_i\}} \exp\left(X_j^\top \beta_T + V_j\right)} + \begin{pmatrix} 0_{\mathrm{g}} \\ \sigma_e^2 \beta_{Tm} \end{pmatrix}\right] \tag{S22}$$

Solving the equation $\Psi_c^*\left(R_i, T_i, \delta_i, \mathbf{Z}_i, TMB_i^*; \mathbf{\Theta}\right) = 0$, the approximately consistent estimators $\tilde{\theta}$, $\tilde{u}_i$ and $\tilde{v}_i$ can be derived.

2 SIMULATIONS

2.1 Simulation study without considering TMB measurement errors

In order to assess the proposed estimation procedure's performance, we conducted a series of simulation studies whose primary objective was to assess the fixed effect estimates and the variance of the random effects. Data are simulated in an oncology trial context, with random effects correlated among clusters' multiple endpoints. In the simulations, we assume 200 patients, i.e., $i = 1, ..., 200$. For each cluster i, we generate the random effects U_{1i}, U_{2i} and V_i from a multivariate normal distribution with zero mean, $\theta_1 = \theta_2 = \theta_3 = 1.0$, and general correlations ρ_1, ρ_2, ρ_3. We consider three distinct tumor response states CR&PR ($k = 0$ is being setting as the reference), SD ($k = 1$) and PD ($k = 2$). The response data is generated based on the logistic probabilities for multiple classifications, $\pi_0 = 1 - (\pi_1 + \pi_2)$, $\pi_1 = \exp\eta_1/\left(1 + \exp\eta_1 + \exp\eta_2\right)$, $\pi_2 = \exp\eta_2/\left(1 + \exp\eta_1 + \exp\eta_2\right)$. The event time for the patient is generated from the probability density function $\lambda_0(t)\exp\eta_{3ij}S_0(t)^{\exp\eta_{3ij}}$, where the baseline hazard $\lambda_0(t)$ is assumed to follow the exponential distribution with a mean equal to 500, and $S_0(t) = \exp(-\int_0^t \lambda_0(s)ds)$ is the baseline survival function. Censoring time C is generated from the uniform distribution $U(50, 500)$.

Furthermore, we set $\beta_{R1} = 1.6$, $\gamma_1 = -0.2$, $\beta_{R2} = 1.0$, $\gamma_1 = -0.8$, $\beta_T = -1.2$, $\theta_1 = \theta_2 = \theta_3 = 1.0$. The correlation $\rho_m(m = 1, 2, 3)$ is chosen from -0.9, -0.8, -0.6, -0.4, -0.2, 0, 0.2, 0.4, 0.6, 0.8 and 0.9, in order to evaluate the performance of the estimators with different correlation levels. In the negative correlation scenario simulations, only two of the three values for ρ_m can be set to negative, given the positive definite limitation of the covariance matrix of the multivariate Gaussian distribution. With the specified parameters, 1000 replications are simulated in each setting. Results of the simulations are presented in Table S1. We report the fitted value, average bias, SE and SD for each parameter, where SE and SD are defined as the average of the standard error of the estimates and the standard error of the estimates over 1000 simulations, respectively.

The results in Table S1 indicate that the regression parameter estimates in three function sections perform fairly well under various multivariate correlation settings, given that the hazard estimation is performed using a semi-parametric method. The

biases in estimating the variance component parameters $\theta_1, \theta_2, \theta_3, \rho_1, \rho_2, \rho_3$ are likewise relatively minimal. In general, the results support the suggested multi-endpoint joint model with correlated random effects being applicable. When comparing SE and SD, the accuracy of the reported standard errors is generally acceptable, though slightly underestimated when ρ_m approaches the limiting values of ± 0.9. In particular, the sample standard errors SD for 1000 correlation estimates are minimal due to the limited one-way volatility on correlation estimates at boundaries (i.e., the correlation estimates can only be larger than -1 or smaller than 1). However, the estimation method for standard error still assumes two-way variation. As a result, when ρ_m is close to the limits, bootstrap estimates for the standard error of the correlation parameter could be employed.

Table S1: Bias and standard error of estimators for joint model with multivariate random effects.

Parameter	True value	Fitted value	Average Bias	SE	SD
Simulation 1					
β_{R1}	1.6	1.574	0.026	0.642	0.646
γ_1	-0.2	-0.207	0.007	0.421	0.423
β_{R2}	1.0	0.946	0.054	0.775	0.809
γ_2	-0.8	-0.806	0.006	0.486	0.495
β_T	-1.2	-1.190	0.010	0.484	0.481
θ_1	1.0	1.059	0.059	0.553	0.546
θ_2	1.0	1.114	0.114	0.673	0.638
θ_3	1.0	0.986	0.014	0.450	0.448
ρ_1	0.9	0.859	0.041	0.208	0.152
ρ_2	0.9	0.851	0.049	0.251	0.153
ρ_3	0.9	0.834	0.066	0.308	0.172
Simulation 2					
β_{R1}	1.6	1.543	0.057	0.642	0.621
γ_1	-0.2	-0.184	0.016	0.416	0.394
β_{R2}	1	0.971	0.029	0.772	0.795
γ_2	-0.8	-0.801	0.001	0.482	0.492
β_T	-1.2	-1.212	0.012	0.487	0.478
θ_1	1	0.987	0.013	0.530	0.556
θ_2	1	1.042	0.042	0.648	0.581
θ_3	1	0.976	0.024	0.447	0.449
ρ_1	0.8	0.767	0.033	0.264	0.213
ρ_2	0.8	0.763	0.037	0.287	0.197
ρ_3	0.8	0.725	0.075	0.342	0.233
Simulation 3					
β_{R1}	1.6	1.560	0.040	0.650	0.671
γ_1	-0.2	-0.187	0.013	0.421	0.408
β_{R2}	1	0.998	0.002	0.769	0.767
γ_2	-0.8	-0.776	0.024	0.478	0.478
β_T	-1.2	-1.211	0.011	0.484	0.505
θ_1	1	1.016	0.016	0.544	0.585
θ_2	1	1.005	0.005	0.634	0.599
θ_3	1	0.926	0.074	0.429	0.450
ρ_1	0.6	0.593	0.007	0.349	0.305
ρ_2	0.6	0.596	0.004	0.318	0.293

ρ_3	0.6	0.569	0.031	0.389	0.309
Simulation 4					
β_{R1}	1.6	1.557	0.043	0.650	0.642
γ_1	-0.2	-0.197	0.003	0.416	0.425
β_{R2}	1	0.973	0.027	0.764	0.784
γ_2	-0.8	-0.760	0.040	0.474	0.499
β_T	-1.2	-1.197	0.003	0.486	0.504
θ_1	1	0.914	0.086	0.511	0.501
θ_2	1	0.957	0.043	0.617	0.617
θ_3	1	0.922	0.078	0.428	0.433
ρ_1	0.4	0.386	0.014	0.435	0.375
ρ_2	0.4	0.402	0.002	0.357	0.341
ρ_3	0.4	0.398	0.002	0.420	0.365
Simulation 5					
β_{R1}	1.6	1.549	0.051	0.655	0.667
γ_1	-0.2	-0.185	0.015	0.416	0.413
β_{R2}	1	0.972	0.028	0.764	0.776
γ_2	-0.8	-0.751	0.049	0.471	0.472
β_T	-1.2	-1.185	0.015	0.486	0.491
θ_1	1	0.910	0.090	0.512	0.509
θ_2	1	0.907	0.093	0.601	0.564
θ_3	1	0.912	0.088	0.425	0.439
ρ_1	0.2	0.181	0.019	0.498	0.433
ρ_2	0.2	0.208	0.008	0.381	0.384
ρ_3	0.2	0.187	0.013	0.440	0.411
Simulation 6					
β_{R1}	1.6	1.550	0.050	0.663	0.664
γ_1	-0.2	-0.178	0.022	0.419	0.410
β_{R2}	1	1.021	0.021	0.763	0.761
γ_2	-0.8	-0.737	0.063	0.471	0.465
β_T	-1.2	-1.236	0.036	0.489	0.501
θ_1	1	0.923	0.077	0.522	0.524
θ_2	1	0.927	0.073	0.609	0.578
θ_3	1	0.904	0.096	0.422	0.438
ρ_1	0	0.027	0.027	0.530	0.433
ρ_2	0	0.003	0.003	0.385	0.386
ρ_3	0	-0.002	0.002	0.440	0.419
Simulation 7					
β_{R1}	1.6	1.601	0.001	0.669	0.655
γ_1	-0.2	-0.183	0.017	0.421	0.408
β_{R2}	1	1.063	0.063	0.766	0.759
γ_2	-0.8	-0.746	0.054	0.472	0.480
β_T	-1.2	-1.201	0.001	0.489	0.498
θ_1	1	0.929	0.071	0.528	0.526
θ_2	1	0.921	0.079	0.611	0.582
θ_3	1	0.917	0.083	0.428	0.436
ρ_1	-0.2	-0.153	0.047	0.574	0.439
ρ_2	-0.2	-0.196	0.004	0.381	0.377

ρ_3	0.2	0.188	0.012	0.444	0.407
Simulation 8					
β_{R1}	1.6	1.612	0.012	0.674	0.692
γ_1	-0.2	-0.193	0.007	0.425	0.431
β_{R2}	1	1.060	0.060	0.764	0.745
γ_2	-0.8	-0.731	0.069	0.470	0.465
β_T	-1.2	-1.208	0.008	0.486	0.519
θ_1	1	0.950	0.050	0.539	0.522
θ_2	1	0.916	0.084	0.609	0.586
θ_3	1	0.903	0.097	0.421	0.420
ρ_1	-0.4	-0.298	0.102	0.582	0.419
ρ_2	-0.4	-0.383	0.017	0.366	0.332
ρ_3	0.4	0.370	0.030	0.425	0.372
Simulation 9					
β_{R1}	1.6	1.584	0.016	0.678	0.681
γ_1	-0.2	-0.168	0.032	0.427	0.421
β_{R2}	1	1.001	0.001	0.766	0.792
γ_2	-0.8	-0.691	0.109	0.473	0.492
β_T	-1.2	-1.227	0.027	0.484	0.504
θ_1	1	0.954	0.046	0.545	0.548
θ_2	1	0.948	0.052	0.623	0.584
θ_3	1	0.939	0.061	0.433	0.441
ρ_1	-0.6	-0.468	0.132	0.591	0.366
ρ_2	-0.6	-0.580	0.020	0.338	0.289
ρ_3	0.6	0.553	0.047	0.387	0.318
Simulation 10					
β_{R1}	1.6	1.601	0.001	0.685	0.673
γ_1	-0.2	-0.164	0.036	0.429	0.427
β_{R2}	1	1.055	0.055	0.771	0.788
γ_2	-0.8	-0.711	0.089	0.476	0.500
β_T	-1.2	-1.222	0.022	0.483	0.502
θ_1	1	0.955	0.045	0.549	0.559
θ_2	1	0.980	0.020	0.637	0.626
θ_3	1	0.950	0.050	0.436	0.452
ρ_1	-0.8	-0.627	0.173	0.589	0.302
ρ_2	-0.8	-0.758	0.042	0.309	0.215
ρ_3	0.8	0.727	0.073	0.343	0.246
Simulation 11					
β_{R1}	1.6	1.634	0.034	0.689	0.693
γ_1	-0.2	-0.169	0.031	0.432	0.421
β_{R2}	1	1.061	0.061	0.775	0.781
γ_2	-0.8	-0.728	0.072	0.481	0.492
β_T	-1.2	-1.194	0.006	0.482	0.496
θ_1	1	0.985	0.015	0.562	0.533
θ_2	1	1.039	0.039	0.662	0.641
θ_3	1	0.980	0.020	0.447	0.458
ρ_1	-0.9	-0.730	0.170	0.567	0.266
ρ_2	-0.9	-0.830	0.070	0.288	0.177

ρ_3	0.9	0.827	0.073	0.313	0.185

2.2 Simulation study considering TMB measurement errors

In order to assess the performance of the proposed joint model with corrected-score function, we also conducted a series of simulation studies under TMB measurement errors. The simulation setting is similar with the above section 2.1. $\beta_{Rz} = -1.8$, $\beta_{Rm} = 0.3$, $\beta_{Tz} = 2.2$, $\beta_{Tm} = -0.4$. The variance for the error term is set to be 0.5, 0.75, 1.0, respectively, in order to evaluate the performance of the estimators with different measurement error levels. With the specified parameters, for each dataset, based on joint model, the true-data estimator, the naive estimator and the correct-score estimator with Monte Carlo approximation J=10, were computed 1000 replications. As a comparison, we also based the standard regression models, the true-data estimator and the naive estimator were computed. Results of the simulations are presented in Table S2. We report the fitted value, average bias, SD and SE for each parameter, where SD and SE are defined as the standard error of the estimates over 1000 simulations and the average of the standard error of the estimates, respectively.

According to Table S2, the regression parameter estimates for the two function components perform reasonably well for a variety of measurement error conditions. In the absence of measurement errors, the joint model outperforms ordinary regression models in calculating regression coefficients because it more precisely reflects the potential connections between several endpoints. When considering different levels of measurement errors, the performance of the estimator based on corrected-score was significantly superior to that of the naive estimator and only marginally poorer than that of the true-data estimator. Clearly, the performance of the naive estimator deteriorates with increasing error magnitude, which further suggests that measurement error introduces a more significant bias effect on the parameter estimates. Overall, the results of the simulation experiments support the proposed joint multi-endpoint model and the iterative numerical estimation procedure, as well as the applicability of the associated random effects. Additionally, comparing SE and SD, the precision of the stated standard errors is generally satisfactory. The biases of the joint assessments compared to the standard separate regressions emphasizes that the dependence among clinical endpoints could be an important and non-negligible con-founder in analyzing the factors determining the treatment effect.

Table S2: Bias and standard error of estimators for joint model considering varying measurement errors.

Parameter	True value	Fitted value	Average Bias	SE	SD
Correct-score $\sigma_e = 0.5$					
β_{Rz}	-1.8	-1.854	0.054	0.503	0.495
β_{Rm}	0.3	0.278	0.022	0.092	0.091
β_{Tz}	2.2	2.117	0.083	0.296	0.373
β_{Tm}	-0.4	-0.425	0.025	0.06	0.081
Correct-score $\sigma_e = 0.75$					
β_{Rz}	-1.8	-1.798	0.002	0.502	0.477
β_{Rm}	0.3	0.271	0.029	0.091	0.09
β_{Tz}	2.2	2.108	0.092	0.293	0.37
β_{Tm}	-0.4	-0.418	0.018	0.059	0.076
Correct-score $\sigma_e = 1.0$					
β_{Rz}	-1.8	-1.79	0.01	0.498	0.444
β_{Rm}	0.3	0.274	0.026	0.09	0.081
β_{Tz}	2.2	2.027	0.173	0.288	0.362
β_{Tm}	-0.4	-0.39	0.01	0.058	0.074
Naive estimator $\sigma_e = 0.5$					

β_{Rz}	-1.8	-1.685	0.115	0.482	0.409
β_{Rm}	0.3	0.257	0.043	0.087	0.069
β_{Tz}	2.2	1.757	0.443	0.281	0.326
β_{Tm}	-0.4	-0.344	0.056	0.056	0.06
Naive estimator $\sigma_e = 0.75$					
β_{Rz}	-1.8	-1.591	0.209	0.465	0.403
β_{Rm}	0.3	0.246	0.054	0.082	0.066
β_{Tz}	2.2	1.436	0.764	0.264	0.296
β_{Tm}	-0.4	-0.268	0.132	0.051	0.047
Naive estimator $\sigma_e = 1.0$					
β_{Rz}	-1.8	-1.585	0.215	0.456	0.483
β_{Rm}	0.3	0.224	0.076	0.079	0.078
β_{Tz}	2.2	1.335	0.865	0.255	0.376
β_{Tm}	-0.4	-0.26	0.14	0.048	0.082

3 COHORT INFORMATION

3.1 Patient characteristics

Radiographic tumor assessments were taken at baseline and approximately every 6 weeks. ORR was the proportion of patients with CR or PR. Treatment continued until confirmed PD, intolerable toxicities, death or withdrawal of consent. Censored data documented the last radiographic assessment before cut-off, loss of follow-up, or change of treatment. Progression-free survival (PFS) was defined as the time from the first dose to PD or prior death. Overall survival (OS) was duration from the first dose to death, and patients who remained alive were censored at the date of their last follow-up. This study was conducted in accordance with the Declaration of Helsinki and was approved by the Ethical Review Committee of Sun Yat-sen University Cancer Center. Written informed consents were provided by all patients. Tumor samples from all NPC patients were analyzed using both WES and Panel. FFPE samples were consistently used in the analysis due to the paucity of biopsy samples and given the limited intra-tumor heterogeneity they represent. The clinical characteristics of this cohort are summarized in Table 1 with details in Supplementary Table S1. For lung cancer, 60% of the patients had adenocarcinoma, followed by squamous carcinoma (31%). Almost all patients (99%) were stage IV at diagnosis; the median ages of patients with NSCLC and NPC at the treatment initiation were 54 and 46 years, respectively. 49% of the NSCLC patients and 25% of the NPC patients had a smoking history, and there were more males in both cohorts (68% vs. 32% for NSCLC, 80% vs. 20% for NPC). ORR of the study cohorts was 18% and 12%. The median progression-free survival (mPFS) for lung cancer and nasopharyngeal carcinoma was 60 and 67.5 days, respectively.

3.2 Library preparation and sequencing

For whole-exome sequencing (WES), genomic DNAs were extracted from Formalin-fixed, paraffin-embedded (FFPE) or biopsy tumor and blood samples by QIAamp DNA FFPE Tissue Kit and DNeasy Blood & Tissue Kit (Qiagen), respectively, and quantified by Qubit 3.0 using the dsDNA HS Assay Kit (ThermoFisher Scientific). Library preparations were performed with KAPA Hyper Prep Kit (KAPA Biosystems). Target enrichment was performed using the xGen Exome Research Panel and Hybridization and Wash Reagents Kit (Integrated DNA Technology). Sequencing was performed on Illumina HiSeq4000 platform using PE150 sequencing chemistry (Illumina). For targeted-panel, customized xGen lockdown probes (Integrated DNA Technologies) targeting 422 cancer-relevant genes were used for hybridization enrichment. The capture reaction was performed with Dynabeads M-270 (Life Technologies) and xGen Lockdown Hybridization and Wash Kit (Integrated DNA Technologies). Captured libraries were on-beads PCR amplified with Illumina p5 and p7 primers in KAPA HiFi HotStart ReadyMix (KAPA Biosystems), followed by purification using Agencourt AMPure XP beads. Libraries were quantified by qPCR using KAPA Library Quantification Kit (KAPA Biosystems). Library fragment size was determined by Bioanalyzer 2100 (Agilent Technologies). The target-enriched library was then sequenced on HiSeq4000 or HiSeq4000 NGS platforms

(Illumina). The average coverage depth was 140X and 1341X for tumors (64X and 143X for normal controls) using WES and Panel, respectively.

3.3 Bioinformatics procedure for sequencing data analysis

FASTQ files were processed using Trimmomatic for quality control. Leading/trailing low quality (below 20) or N bases were removed. The high quality paired-end reads were aligned to the human hg19 reference genome using the Burrows-Wheeler Aligner. The resulting alignment files were cleaned by Picard and standardized GATK3, including deduplication, BQSR and Indel realignment. Cross-sample contamination was estimated using ContEst (Broad Institute, contamination rate ¡0.02). Somatic single nucleotide variant (SNV) and insertion/deletions (indels) calling was performed by Mutect and Scalpel, respectively. Variants called were further filtered using the following criteria: (i) 4 reads and 2% variant allele frequency (VAF) supporting the variant, (ii) filtered if present in ¿1% population frequency in the 1,000 g or ExAC database, (iii) filtered through an internally collected list of recurrent sequencing errors (3 variant reads and 20% VAF in at least 30 out of 2,000 normal samples) on the same sequencing platform. Final list of mutations was annotated using vcf2maf. Tumor mutation burden (TMB) for WES was defined as the total number of missense mutations. In addition, we also used a targeted next-generation sequencing panel (Geneseeq) to profile TMB of these samples and compare it to WES results. Panel TMB was calculated by adding up all base substitutions and indels in the coding region of targeted genes. Synonymous variants were counted to reduce sampling noise, and known driver mutations were excluded due to over-representation, as described in previous study.
